# Activated carbon synthesised from lignocellulosic cocoa pod husk *via* alkaline and acid treatment for methylene blue adsorption: optimisation by response surface methodology, kinetics, and isotherm modelling

**DOI:** 10.1039/d5ra05557a

**Published:** 2025-12-01

**Authors:** Zenaida Guerra-Que, Katia S. López-Margalli, Juan Manuel Urrieta-Saltijeral, Adib Abiu Silahua-Pavón, Héctor Martínez-García, Pedro García-Alamilla, Gerardo E. Córdova-Pérez, Juan Carlos Arévalo-Pérez, José Gilberto Torres-Torres

**Affiliations:** a Tecnológico Nacional de México-Instituto Tecnológico de Villahermosa (TECNM – I. T. Villahermosa), Laboratorio de Investigación 1 Área de Nanotecnología Km. 3.5 Carretera Villahermosa–Frontera, Cd. Industrial C.P. 86010 Villahermosa Tabasco Mexico zenaida.gq@villahermosa.tecnm.mx zenaida.guerra4@gmail.com +52 9933046111; b División Académica de Ciencias Agropecuarias (DACA), Universidad Juárez Autónoma de Tabasco (UJAT) Carret. Villahermosa-Teapa Km 25, Ra. La Huasteca Tabasco, 86280 Mexico; c Benemérita Universidad Autónoma de Puebla, Facultad de Ciencias químicas, Centro Avanzado de Pruebas Analíticas no Destructivas 72570 Puebla Mexico; d Universidad Juárez Autónoma de Tabasco, Centro de Investigación de Ciencia y Tecnología Aplicada (CICTAT), DACB, Laboratorio de Nanomateriales Catalíticos Aplicados al Desarrollo de Fuentes de Energía y Remediación Ambiental Km.1 carretera Cunduacán-Jalpa de Méndez Cunduacán Tabasco, C.P. 86690 Mexico

## Abstract

This study reports the green synthesis of activated carbon (ANC) from Cocoa Pod Husk (CPH), a lignocellulosic agrowaste, using sulphuric acid and sodium hydroxide treatments for Methylene Blue (MB) removal. Among the synthesised samples, ANCSH5 (NaOH, 5 M) exhibited superior performance due to extensive delignification, enhanced graphitization, and an abundance of oxygenated functionalities, as confirmed by Fourier Transform Infrared (FTIR), Scanning Electron Microscopy – Energy Dispersive X-ray (SEM–EDX), the Brunauer–Emmett–Teller Method (BET), X-ray Diffraction (XRD), Point of Zero Charge (pH_PZC_), and Raman analyses. ANCSH5 achieved 92% MB removal (1.16 mg g^−1^), surpassing CPH (24%, 0.30 mg g^−1^) and ANCSA10 (44%, 0.50 mg g^−1^). Adsorption followed pseudo-second-order kinetics, while equilibrium data fitted best to the Freundlich, Sips, Toth, and Redlich–Peterson models (*R*^2^ > 0.999). Process optimisation *via* a central composite design (CCD) and Response Surface Methodology (RSM) determined optimal conditions (49.6 °C, pH 6.0), achieving a 99.05% removal rate. Importantly, isotherm modelling predicted a competitive *q*_max_ of 100 mg g^−1^ for ANCSH5 under practical conditions. Unlike conventional biomass carbons, the hemicellulose-rich CPH precursor promoted extensive delignification, partially graphitised domains, and tailored surface chemistry, providing a distinctive adsorption mechanism. These features underscore CPH valorisation as a sustainable route to high-performance, low-cost adsorbents for dye remediation in cocoa-producing regions.

## Abstract 2

Dual activation of cocoa pod husk yields carbon with competitive adsorption of methylene blue, integrating response surface optimisation for sustainable wastewater remediation.

## Graphical abstract

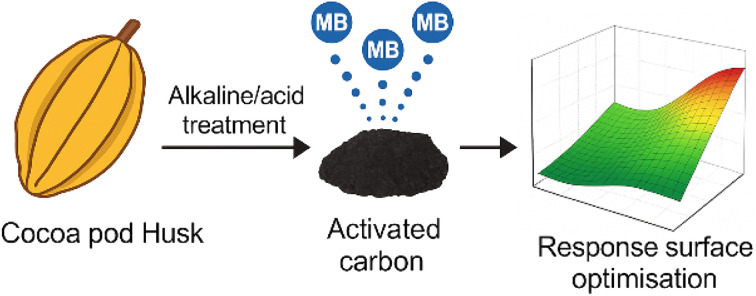

## 1 Introduction

Access to clean and safe water has become a critical global challenge, as less than 1% of the earth's total water supply is available as freshwater from rivers, lakes, and aquifers.^1^ Industrial discharges, particularly from the textile, paper, and leather industries, contribute significantly to environmental degradation and pose risks to both human health and aquatic ecosystems.^2–5^ Among the priority pollutants, synthetic dyes have been classified as persistent organic pollutants (POPs) under the Stockholm Convention, owing to their high chemical stability, toxicity, and poor biodegradability.^6–8^

**Fig. 1:**
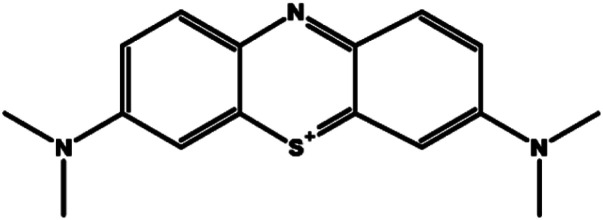
Molecular structure of MB dye.

Methylene blue (MB) is a widely used cationic dye in textile and paper processing, whose release into aquatic environments can cause carcinogenic, mutagenic, and ecotoxic effects.^9–11^ Due to its complex aromatic structure (Fig. 1), MB is resistant to conventional treatment methods (biological or physicochemical), which are often inefficient or economically unsustainable.^12,13^ In this context, adsorption onto Activated Carbon (AC) has emerged as a cost-effective and efficient alternative compared to other technologies such as ion exchange, membrane filtration, or photocatalysis.^13–15^

In developing countries such as Mexico, wastewater from the textile industry is often discharged directly into rivers and other natural water bodies, either untreated or insufficiently treated.^16,17^ This practice significantly contributes to the deterioration of aquatic ecosystems and highlights the urgent need for cost-effective and sustainable wastewater treatment technologies.

Cocoa Pod Husk (CPH), an abundant agro-industrial waste in cocoa-producing regions such as Mexico, represents a largely underutilised precursor for carbon production. For every ton of dry cocoa beans, approximately 10 tons of CPH are discarded, generating a significant environmental burden.^18–24^ According to the Mexican Agriculture Public Organization (MAPO, 2022),^25^ about 28 000 tonnes of cocoa beans were produced in 2021, corresponding to nearly 280 000 tonnes of CPH waste. The lignocellulosic composition of CPH, rich in hemicellulose, lignin, and cellulose, makes it an attractive candidate for chemical activation and valorisation into carbon-based nanomaterials.^18–21^ Unlike other biomass sources,^26–32^ the high hemicellulose fraction of CPH facilitates delignification, enabling the formation of nanocarbon domains with distinct surface functionalities.

In particular, CPH has been reported as a promising feedstock in recent studies, confirming its potential for removing organic pollutants from water.^21,23,33,34^ The development of carbon-based materials has also been highlighted in other contexts, such as polymeric composites and structural applications, demonstrating the versatility and broad applicability of carbon materials.^35^ Nevertheless, limitations remain regarding pore efficiency under variable operating conditions. Moreover, few studies have applied robust statistical design approaches such as Response Surface Methodology (RSM) to optimise critical process parameters simultaneously.

In this work, we applied two independent chemical activation strategies, alkaline (NaOH) and acidic (H_2_SO_4_), to CPH, a non-conventional lignocellulosic precursor, to synthesise Activated Carbon (ANC). Unlike conventional biomass precursors such as rice husk or coconut shell, CPH possesses a uniquely high hemicellulose-to-lignin ratio, which enables more extensive delignification and the development of partially graphitised domains enriched with oxygen-containing functionalities.^18,20,36,37^ These distinctive structural and chemical transformations differentiate CPH-derived ANC from previously reported biomass-based carbons, providing higher graphitization and enhanced adsorption performance.^38–41^ The resulting materials were comprehensively characterised to elucidate surface chemistry and textural evolution, while adsorption toward MB was evaluated through kinetic and equilibrium modelling. Furthermore, process optimisation was performed using central composite design (CCD) and RSM, a method that has not been systematically applied to CPH-derived carbons. Beyond performance, this study highlights the green valorisation of an abundant agro-waste into chemically activated adsorbents, aligning with circular economy principles. By coupling enhanced graphitisation with optimised adsorption efficiency, the proposed approach highlights the green valorisation of an abundant agro-waste into chemically activated adsorbents, aligning a sustainable and cost-effective solution for wastewater remediation in cocoa-producing regions.

## 2 Methods

### 2.1 Precursor material

The CPH utilized in this study were collected from the farm of Mr Efrén Hernández Maldonado, located in Ra. Mihuatlán, Cunduacán, Tabasco, México. The farm supplies raw materials to Chocolatería Artesanal Citlalli, owned by Mr Cutberto Lázaro Cepeda.

The raw material, referred to as CPH, comprised 4 kg of agricultural waste. The husks were thoroughly washed multiple times with deionized water to remove dust and surface impurities. Subsequently, the material was air-dried under sunlight for three days on a flat, open surface, followed by oven drying at 100 °C for 48 hours using a Riossa oven (Riossa, Mexico). After drying, the material was ground and sieved to obtain particles smaller than 0.25 mm (mesh size 60–80), using a conventional mechanical sieve shaker. Similar pre-treatment steps have been widely reported for lignocellulosic precursors to ensure homogeneity and surface cleanliness prior to activation.^15,23,24^ The resulting CPH powder was subjected to the determination of extractives, cellulose, hemicellulose, and lignin contents following the standard analytical protocols reported in the Official Methods of Analysis of AOAC International (18th ed.),^42^ in accordance with the acid–alkali digestion and solvent extraction procedures commonly employed for lignocellulosic residues [Sluiter *et al.*, NREL/TP-510-42618, 2012].^43^ Comparable pre-treatment and characterisation methodologies for lignocellulosic precursors have also been reported by Selvaraju and Bakar (2017)^44^ and Li *et al.* (2014),^45^ whose studies are widely cited and validate the reliability of these analytical techniques.

### 2.2 Lignocellulosic characterisation of cocoa pod husk (CPH) before treatment

**Fig. 2:**
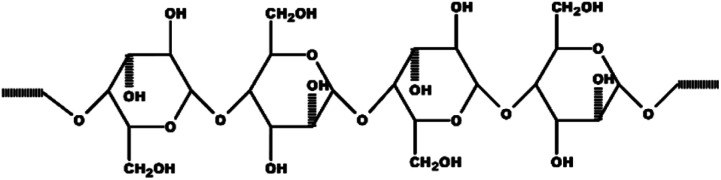
Molecular structure of cellulose, representative of lignocellulosic components in CPH.

The structural composition of lignocellulosic biomass, particularly the arrangement of cellulose, hemicellulose, and lignin, plays a crucial role in determining the reactivity of the precursor and its subsequent activation performance. For clarity, the molecular structure of cellulose is shown in Fig. 2 as a representative component of the lignocellulosic framework in CPH.

#### 2.2.1 Determination of extractives content

The Cocoa Pod Husk Powder (CPHP, *W*_0_) was mixed with a benzene/ethanol solution (2 : 1 v/v) and maintained at a constant temperature for 3 hours to extract soluble components. The mixture was then filtered, and the solid residue was dried in an oven at 105 °C for 24 hours until a constant weight was achieved. After cooling to room temperature in a desiccator, the sample was weighed (*W*_1_). The extractable content (% Ext) was calculated using eqn (1):1
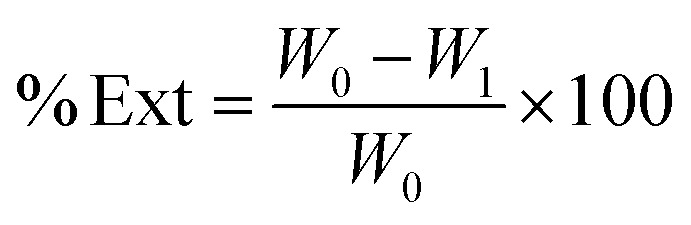


The remaining residue was subsequently used for quantifying the hemicellulose and lignin contents.^42–45^

#### 2.2.2 Determination of hemicellulose content

A volume of 150 mL of sodium hydroxide solution (20 g L^−1^) was added to the extractive-free residue (*W*_2_). The mixture was placed in a covered beaker and heated at a constant temperature for 3.5 hours. The resulting solid was separated by Büchner filtration and washed four times with 150 mL of distilled water to remove residual sodium ions. The solid was then dried in an oven at 105 °C for 24 hours and weighed (*W*_3_).^42–45^ The hemicellulose content (% Hemi) was determined using eqn (2):2
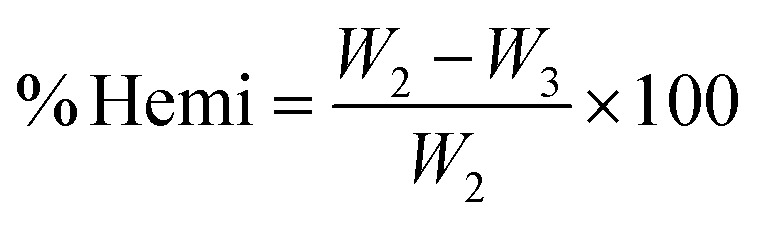


#### 2.2.3 Determination of lignin content

A 1 g portion of the residue obtained from the extractive analysis was dried in an oven at 105 °C until constant weight and then cooled in a desiccator before being weighed (*W*_4_). Subsequently, 30 mL of concentrated sulphuric acid (72% H_2_SO_4_) was slowly added to the sample. The mixture was maintained at a temperature between 8 and 15 °C for 24 hours. After this period, the sample was transferred to a flask, diluted with 300 mL of distilled water, and heated to a boil for 1 hour. The resulting residue was filtered, thoroughly washed to remove residual sulfate ions, dried at 50 °C to constant weight, cooled in a desiccator, and finally weighed (*W*_5_).^42–45^ The lignin content (% Lignin) was calculated using eqn (3):3
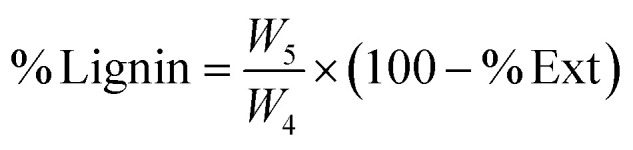


#### 2.2.4 Determination of cellulose content (by difference)

The cellulose content (% Cellulose) was determined by difference, using eqn (4):

4 % Cellulose = 100 − % Ext − % Hemi − % Lignina

#### 2.2.5 Proximate analysis of CPHP

Proximate analysis was carried out by the official methods of the Association of Official Analytical Chemists (AOAC, 2005),^42^ including moisture content (Method 925.09), crude fat (920.30), crude protein (979.09), ash (923.03), and crude fiber (962.09). Carbohydrate content was calculated by difference.

### 2.3 Green synthesis of ANC from CPH powder

The delignification process for material synthesis was conducted *via* immersion in alkaline and acidic solutions. Treatments were carried out using a 1 : 10 (w/v) ratio of lignocellulosic residue to chemical solution at room temperature for 12 hours, with two concentration levels applied for each condition. For alkaline activation, CPHP, previously oven-dried, was immersed in sodium hydroxide solutions at concentrations of 0.1 M and 5 M (solid-to-liquid ratio of 100 g L^−1^). The resulting samples were designated as ANCSH0.1 and ANCSH5, respectively. Similarly, acidic treatment was performed by immersing CPHP in sulphuric acid solutions at 0.5 M and 10 M concentrations, yielding samples labelled ANCSA0.5 and ANCSA10. After treatment, all suspensions were vacuum-filtered and thoroughly washed with deionised water until the filtrate reached a neutral pH, followed by rinsing with acetone. The treated solids were then oven-dried at 80 °C until constant weight was achieved, typically within 24 hours. Previous studies have demonstrated the effectiveness of alkali and acid pretreatments in modifying CPH for enhanced dye adsorption, particularly sodium hydroxide delignification to improve surface reactivity and porosity.^20,23,24,33,34,36^

#### 2.3.1 Characterisation of ANC synthesised from CPH

##### 2.3.1.1 N_2_ adsorption–desorption isotherm

The specific surface area, pore diameter, and pore volume of the CPH and ANC samples were determined *via* N_2_ physisorption using a Micromeritics TriStar II 3020 surface area analyzer at 77 K (−196 °C). Before analysis, approximately 0.1 g of each sample was degassed at 300 °C for 3 hours to remove adsorbed impurities and moisture. The obtained adsorption–desorption isotherms were analyzed using the Brunauer–Emmett–Teller (BET) method to calculate the specific surface area (*S*_BET_), while pore volume (*P*_V_) and average pore diameter (*P*_S_) were determined using the Barrett–Joyner–Halenda (BJH) model as implemented in the ASAP 2020 software package.^32,36,41^

##### 2.3.1.2 Scanning electron microscopy (SEM) and energy-dispersive X-ray spectroscopy (EDX)

SEM was conducted using a JEOL/EO JSM-6610 microscope operated at 5–20 kV and equipped with EDX. Before imaging, samples were sputter-coated with a thin layer of gold to enhance surface conductivity. A small amount of powdered sample was mounted on carbon adhesive tape fixed to aluminum stubs, followed by gold coating. Micrographs were acquired at magnifications ranging from 100× to 100 00× to examine surface morphology and elemental distribution, which are critical parameters in evaluating biomass-derived carbons.^46^ The characterisation was conducted at the Center for Research in Science and Applied Technology of Tabasco (CICTAT), Academic Division of Basic Sciences, Universidad Juárez Autónoma de Tabasco (UJAT), in collaboration with the Faculty of Chemical Sciences at Benemérita Universidad Autónoma de Puebla (BUAP), with technical support from Dr Gerardo Enrique Córdova Pérez.

##### 2.3.1.3 X-Ray diffraction (XRD)

XRD analysis was performed to identify the crystalline phases and estimate the crystallite sizes of the CPH and ANC samples. Measurements were conducted using a Bruker D2 PHASER diffractometer equipped with a Co Kα radiation source (*λ* = 0.179 nm). Diffraction patterns were collected over a 2*θ* range of 20° to 80° with a total scan time of 660 seconds. Phase identification was performed using the JADE 6 software and the corresponding database. The interpretation of structural ordering in carbonaceous materials through XRD is consistent with previously reported studies on lignocellulosic biomass-derived carbons.^47,48^

The average crystallite size was estimated using the Scherrer eqn (5).5
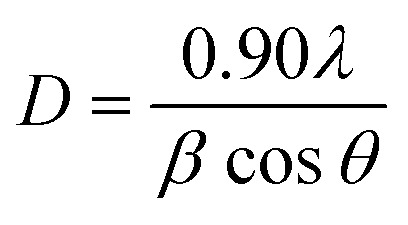
where *λ* represents the X-ray wavelength, *β* the full width at half maximum (FWHM), and *θ* the Bragg angle. This approach has been widely applied for the estimation of crystalline domain sizes in biomass-derived carbons and related disordered carbon materials.^47,49^

##### 2.3.1.4 Raman spectroscopy

Raman spectroscopy was employed to identify vibrational modes in CPH and ANC samples. The measurements were conducted using a HORIBA Xplora Plus Raman microscope equipped with a He–Ne laser (*λ* = 632.8 nm, 16 mW). The system featured spatial resolutions of 20, 5, and 2 µm, and a spectral resolution ranging from ±0.2 to ±0.5 cm^−1^. The instrument was equipped with a thermoelectrically cooled CCD detector, an optical microscope with objectives of 10×, 50×, and 100×, and an integrated video camera for sample focusing. A motorised sample stage enabled mapping over a maximum area of 5 mm^2^. Raman spectroscopy is widely recognised as a powerful tool for probing structural order, bonding, and defect density in carbon-based materials, as extensively discussed in both classical and recent studies.^48,50^

##### 2.3.1.5 Fourier Transform Infrared Spectroscopy (FTIR)

FTIR spectroscopy was performed using a PerkinElmer Frontier spectrophotometer (USA) equipped with a Diamond Attenuated Total Reflection (ATR) accessory and operated *via* Windows®-based software. Spectra were recorded in the wavenumber range of 400–4000 cm^−1^ with a resolution of 4 cm^−1^, averaging 32 scans per spectrum. Samples analysed included untreated CPH, CPH after acid and alkaline treatments, and the corresponding ANC materials. Each spectrum was subjected to baseline correction, smoothing, and normalization using PerkinElmer Spectrum software. All measurements were performed in triplicate, and the resulting spectral data were processed and visualised using OriginPro 8.5.1. FTIR spectroscopy has been widely employed for identifying functional groups and monitoring surface modifications in biomass-derived carbons, providing key insights into chemical transformations during acid and alkaline pretreatments.^28,46^

##### 2.3.1.6 Point of zero charge (pH_PZC_) determination

The pH_PZC_ of the adsorbents was determined using the pH-drift method. In brief, 50 mL of 0.01 M NaCl solution was placed in a series of closed Erlenmeyer flasks, and the initial pH (pH_i_) of each solution was adjusted to values between 2.0 and 12.0 using 0.1 M HCl or 0.1 M NaOH. A fixed mass of adsorbent (0.05 g) was then added, and the suspensions were shaken at room temperature for 24 hours to reach equilibrium. The final pH (pH_f_) of each solution was recorded, and the difference (ΔpH = pH_f_ − pH_i_) was plotted as a function of pH_i_. The pH at which ΔpH = 0 was taken as the pH_PZC_, representing the condition at which the adsorbent surface exhibits no net charge. This procedure has been extensively used for biomass-derived carbons to elucidate surface charge properties and their role in dye adsorption.^38,51–53^

### 2.4 Adsorption experiments

The adsorption performance of the five adsorbent samples, CPH, ANCSH0.1, ANCSH5, ANCSA0.5, and ANCSA10, was evaluated through batch adsorption studies using MB as a model pollutant. Experimental conditions (solid–liquid ratio, initial concentration range, pH window, contact time, and temperature) followed widely adopted protocols for MB adsorption on biomass-derived activated carbons,^14,40^ including CPH-based systems.^24^ For isotherm studies, 400 mg of each adsorbent was added to 100 mL of MB solution at an initial concentration of 5 to 30 mg L^−1^ and pH 6. The effects of contact time (30–180 min), temperature (35–65 °C), and pH (2–6) on MB removal were systematically assessed in line with prior reports on MB adsorption using plant-waste-derived carbons.^51,54–56^ After each adsorption run, samples were vacuum-filtered using 1.2 µm glass microfiber filters. In kinetic experiments, MB concentration was monitored at defined time intervals to resolve uptake profiles, enabling pseudo-order rate modelling and subsequent parameter estimation as commonly applied for MB on hydrochars and agrowaste carbons.^5,57^ MB concentrations before and after adsorption were quantified by UV-Vis spectroscopy (Agilent Technologies, Varian Cary 3000, USA) with *λ*_max_ at 663 nm, consistent with standard MB quantification protocols for lignocellulosic carbons.^51,54–56^ Thermodynamic parameters (Δ*G*°, Δ*H*°, Δ*S*°) were calculated from equilibrium constants (*K*_c_ = *q*_*e*_/*C*_*e*_) using the van't Hoff and Gibbs equations, as described in Section 3.3.3. All experiments were performed in duplicate, and each measurement was performed in triplicate to ensure reproducibility.

The adsorption capacity at equilibrium, *q*_*e*_ (mg g^−1^), was calculated using eqn (6):6
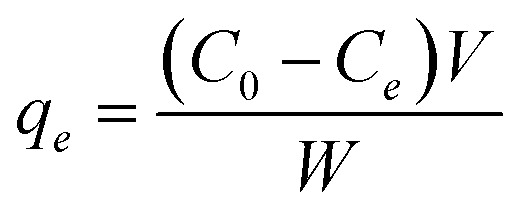
where *C*_0_ is the initial concentration of MB (mg L^−1^); *C*_*e*_ is the concentration of MB at equilibrium (mg L^−1^), *V*, the volume of the solution (L), and *W*, the mass of the absorbent used (g).^14,51^

The following expression was used to calculate the dye removal percentage.7
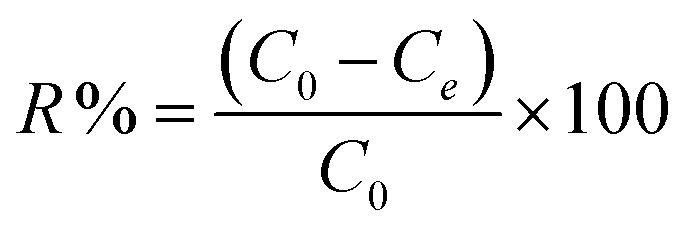


#### 2.4.1 Adsorption kinetics

Exploring adsorption kinetics models provides critical insights into the mechanisms that govern MB uptake onto CPH, ANCSH0.1, ANCSH5, ANCSA0.5, and ANCSA10. Investigating the adsorption rate and the time required to attain equilibrium is essential for evaluating and characterizing the adsorption performance of these materials.^58,59^ In this study, the experimental kinetic data were fitted to two widely used models: the pseudo-first-order and pseudo-second-order kinetic models, to identify the potential rate-limiting steps and gain a better understanding of the adsorption dynamics.

The pseudo-first-order kinetic model^15^ is expressed in linear form in eqn (8).

8 ln(*q*_*e*_ − *q*_*t*_) = ln *q*_*e*_ − *k*_1_*t*

And the nonlinear form is given by ref. 54:

9 *q*_*t*_ = *q*_*e*_(1 − *e*^−*k*_1_*t*^)

where *q*_*e*_ (mg g^−1^) is the amount of adsorbate at equilibrium, *q*_*t*_ (mg g^−1^) is the amount of adsorbate adsorbed at time *t* (min), and *k*_1_ (min^−1^) is the pseudo-first-order rate constant.

The pseudo-second-order kinetic model^60^ in linear form is given by:10
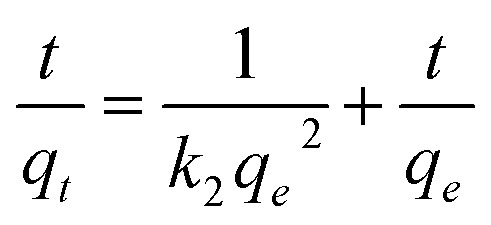
where *k*_2_ (g mg^−1^ min^−1^) is the pseudo-second-order rate constant.

The nonlinear form of the pseudo-second-order kinetic model^61^ is exposed in the following eqn (11).11
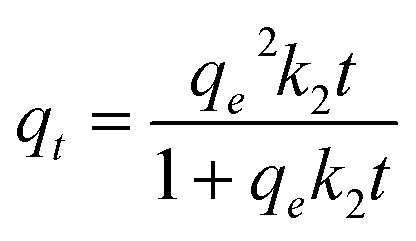


where the variables are the same as in the linear form.

These models assume different adsorption mechanisms: the pseudo-first-order model is typically associated with physisorption. In contrast, the pseudo-second-order model suggests chemisorption involving valence forces through sharing or exchange of electrons between the adsorbent and adsorbate.^41,62^ The data were processed using Origin 8.5 software. By analysing the kinetic parameters and coefficient of determination (*R*^2^), the best-fitting model can be determined, offering valuable insights into the controlling mechanism of the adsorption process.

#### 2.4.2 Isotherm experiments

The adsorption isotherm describes the equilibrium relationship between the amount of adsorbate retained on the adsorbent surface and its concentration remaining in the solution at a constant temperature.^63^ Several adsorption isotherm models are available to analyse experimental data and describe equilibrium adsorption behavior.^64^ In this study, the Langmuir and the Freundlich models were applied to evaluate the adsorption characteristics. In addition to these classical isotherms, the Temkin, Redlich–Peterson, Sips, and Toth models were employed, as they account for surface heterogeneity, energetic variability, and cooperative adsorption effects, particularly relevant when working with bio-based or chemically modified adsorbents.

The Langmuir isotherm^64^ assumes monolayer adsorption onto a surface with a finite number of identical sites and no interaction between adsorbed molecules. It is expressed as:12
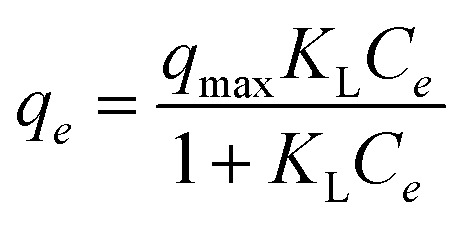
where *q*_*e*_ is the amount of adsorbate adsorbed at equilibrium (mg g^−1^), *q*_max_ is the maximum adsorption capacity corresponding to complete monolayer coverage (mg g^−1^), *C*_*e*_ is the equilibrium concentration of the adsorbate in solution (mg L^−1^), *K*_L_ is the Langmuir equilibrium constant related to the affinity of binding sites (L mg^−1^).

The Freundlich isotherm^54^ is an empirical model that describes adsorption onto heterogeneous surfaces with a non-uniform distribution of heat of adsorption. Its eqn (13) is given by:

13 *q*_*e*_ = *K*_F_*C*^1/*n*^_*e*_

where *q*_*e*_ is the amount of adsorbate adsorbed at equilibrium (mg g^−1^), *K*_F_ is the Freundlich constant indicative of the adsorption capacity (mg^1−1/*n*^ g^−1^ L^1/*n*^), *C*_*e*_ is the equilibrium concentration of the adsorbate in solution (mg L^−1^), *n* is the heterogeneity factor (dimensionless), where values of 1 < *n* < 10 indicate favorable adsorption.

The Redlich–Peterson model^65^ combines the features of both Langmuir and the Freundlich isotherms, offering flexibility for heterogeneous or hybrid adsorption systems. Its general form is:14
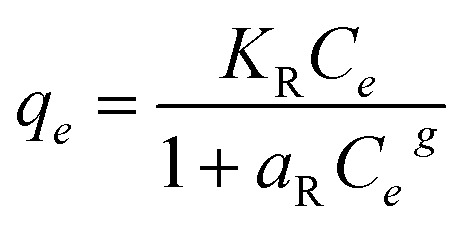
where *q*_*e*_ is the amount of adsorbate adsorbed at equilibrium (mg g^−1^), *K*_R_ (L g^−1^) and *a*_R_ are Redlich–Peterson constants, *C*_*e*_ is the equilibrium concentration of the adsorbate in solution (mg L^−1^), *g* is the heterogeneity factor (dimensionless), with 0 < *g* ≤ 1.

The Sips isotherm^24^ describes adsorption on heterogeneous surfaces and reduces to the Langmuir equation at low concentrations. Its eqn (15) is:15
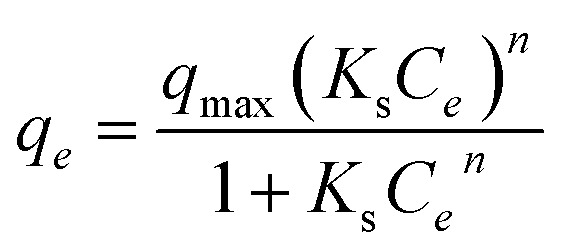
where *q*_max_ is the maximum adsorption capacity (mg g^−1^), *K*_s_ is the Sips isotherm constant related to adsorption intensity (L mg^−1^). *n* is the heterogeneity factor (dimensionless), with *n* = 1 reducing the equation to Langmuir.

The Temkin model^63^ accounts for adsorbate–adsorbent interactions and assumes the heat of adsorption decreases linearly with surface coverage. The eqn (16) is:

16 *q*_*e*_ = *B* ln(*AC*_*e*_)

*A* is the Temkin isotherm equilibrium binding constant (L g^−1^), 
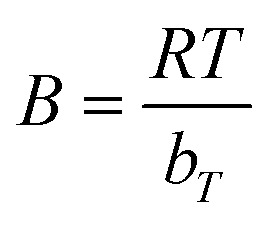
, where *b*_*T*_ is a constant related to the heat of adsorption (J mol^−1^), *R* is the universal gas constant (8.314 J mol^−1^ K^−1^), *T* is the absolute temperature (K).

The Toth isotherm^66^ is an empirical model used to describe adsorption on heterogeneous surfaces. It reduces to Langmuir when the heterogeneity parameter *t* = 1. The eqn (17) is:17
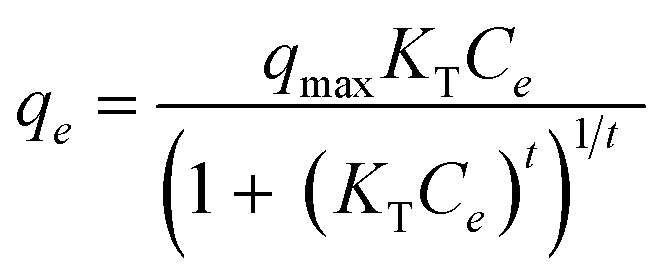


*q*_max_ is the maximum adsorption capacity (mg g^−1^), *K*_T_ is the Toth equilibrium constant (L mg^−1^), *t* is the Toth heterogeneity parameter (dimensionless).

### 2.5 Experimental design and optimisation studies

In this study, a CCD was employed to evaluate the main effects of two experimental variables pH (2–7) and temperature (29–71 °C), as well as their interaction effects on MB adsorption using ANCSH5 as the adsorbent. The Design Expert® software was used to perform the experimental design and RSM analysis. RSM has been widely applied to optimise adsorption processes and evaluate the combined influence of multiple operating variables on dye removal efficiency and adsorption capacity.^67–72^ In particular, RSM and CCD approaches have proven effective in identifying optimal conditions and reducing experimental runs while maintaining statistical robustness, which justifies their application in the present work.^31,67–69^ A CCD comprises factorial points, axial (star) points positioned at a distance *α* from the center, and replicated center points to provide an unbiased estimate of pure error and to test for curvature.^67–69,71,72^ Each factor is evaluated at five coded levels (−*α*, −1, 0, +1, and +*α*), where *α* and the number of experiments (*N*) are calculated using the following equations.

18 *α* = 2^*k*/4^

19 *N* = 2^*k*^ + 2*k* + *C*_p_

where *k* is the number of variables, and *C*_p_ is the number of center point replicates.^67–69^ In many adsorption and related process-optimization studies, the response surface exhibits sufficient curvature that a first-order (linear) model is inadequate; therefore, a second-order polynomial with interaction and quadratic terms is fitted and assessed *via* ANOVA, lack-of-fit, and model adequacy diagnostics.^31,68,69,71,72^ Therefore, to accurately identify critical points (*i.e.*, maxima or minima), a second-order polynomial model is employed, as represented in eqn (20). Quadratic models are widely recognised as essential in RSM studies because they capture surface curvature and enable the prediction of optimal conditions with high reliability.^67–69,71,72^20
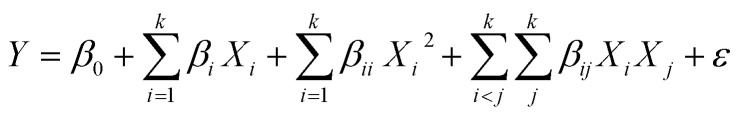
where *Y*, is the predicted response; *X*_*i*_, and *X*_*j*_ are the independent variables; *β*_0_ is the intercept; *β*_*i*_ is the linear coefficient; *β*_*ii*_ is the quadratic coefficient; *β*_*ij*_ is the interaction coefficient between variables *X*_*i*_ and *X*_*j*_; and *ε* is the random error term.

The CCD matrix for this study consisted of 12 experimental runs, including four center points to assess reproducibility and four axial points to ensure rotatability of the design (Table 1). The experimental runs were done at random to minimise systematic error. Analysis of variance (ANOVA) was used to evaluate the significance of individual and interaction effects, with main effects considered statistically significant at *p* < 0.05 (95% confidence level). All experiments were performed in duplicate, with each measurement conducted in triplicate to ensure accuracy and statistical reliability.

**Table 1:** CCD matrix used in the present study

Runs order	Temperature (°C)	pH
1	35	2
2	35	6
3	65	2
4	65	6
5	29	4
6	71	4
7	50	1
8	50	7
9	50	4
10	50	4
11	50	4
12	50	4

Optimisation was carried out using the desirability function to identify the best pH and temperature conditions for maximum MB removal and adsorption capacity.

## 3 Results and discussion

### 3.1 Lignocellulosic characterisation of CPH before treatment

#### 3.1.1 Proximate and lignocellulosic composition analysis of CPH

Table 2 summarises the lignocellulosic fractions, extractables, hemicellulose, lignin, and cellulose, as well as the proximate components, including moisture, protein, fat, ash, fiber, and carbohydrates, in CPHP, expressed as a weight percentage (wt%). Values are reported as mean ± standard deviation.

**Table 2:** Physicochemical and lignocellulosic composition of CPHP

Lignocellulosic composition	Content (wt%)	Proximate analysis	Content (wt%)
Extractable	13.4 ± 0.47	Humidity	9.26 ± 0.02
Hemicellulose	47.73 ± 7.84	Protein	5.29 ± 0.17
Lignin	18.95 ± 9.61	Fat	7.31 ± 0.07
Cellulose	19.92 ± 1.39	Ash (%)	8.03 ± 0.10
		Fiber	23.20 ± 1.36
		Carbohydrate	46.9 ± 0.00

The chemical composition of lignocellulosic bioadsorbents significantly influences their adsorption performance, as it determines the availability of functional groups and surface chemistry. CPHP is primarily composed of natural fiber polymers, with hemicellulose being the most abundant carbohydrate fraction, followed by cellulose and lignin. The cellulose content measured (19.92 wt%) is lower than values reported in other studies,^39,73^ which may be attributed to differences in cocoa variety, cultivation practices, soil characteristics, and regional climatic conditions. In contrast, the hemicellulose content in this study was higher than that reported elsewhere, reinforcing the influence of agricultural and genetic factors on biomass composition.^38,39^

Additional components present in smaller quantities, such as proteins, ashes, and extractables, are grouped under “others” due to their secondary relevance in the structural composition of the biomass. Nevertheless, these constituents may still contribute to the surface reactivity of the material, particularly in chemical activation and adsorption processes.^38^

### 3.2 Characterisation of ANC synthesised from CPH

Table 3 presents the textural parameters of the five adsorbents, determined from the N_2_ adsorption isotherm using the BET method. The specific surface area (*S*_BET_) increases with higher concentrations of chemical treatment, showing a significant rise with sulphuric acid. All five adsorbents are mesoporous in terms of pore size (*P*_s_), with ANCSH0.1 exhibiting the smallest *P*_s_. The chemical treatment removes moisture and volatile matter from the biomass, which typically enhances its reactivity as an adsorbent.^40,74^

**Table 3:** Specific surface area (*S*_BET_), pore volume (*P*_V_), and pore size (*P*_S_) of the adsorbents. N.D. not detected

Adsorbent	*S*_BET_ (m^2^ g^−1^)	*P*_V_ (cm^3^ g^−1^)	*P*_S_ (nm)
CPH	0.0169	0.000778	7
ANCSA0.5	0.129	0.000366	10
ANCSA10	1.966	0.004617	14
ANCSH0.1	0.5115	0.000039	3.5
ANCSH5	0.2218	0.000511	N.D.

#### 3.2.1 Scanning electron microscopy (SEM) with energy dispersive X-ray (EDX)

**Fig. 3:**
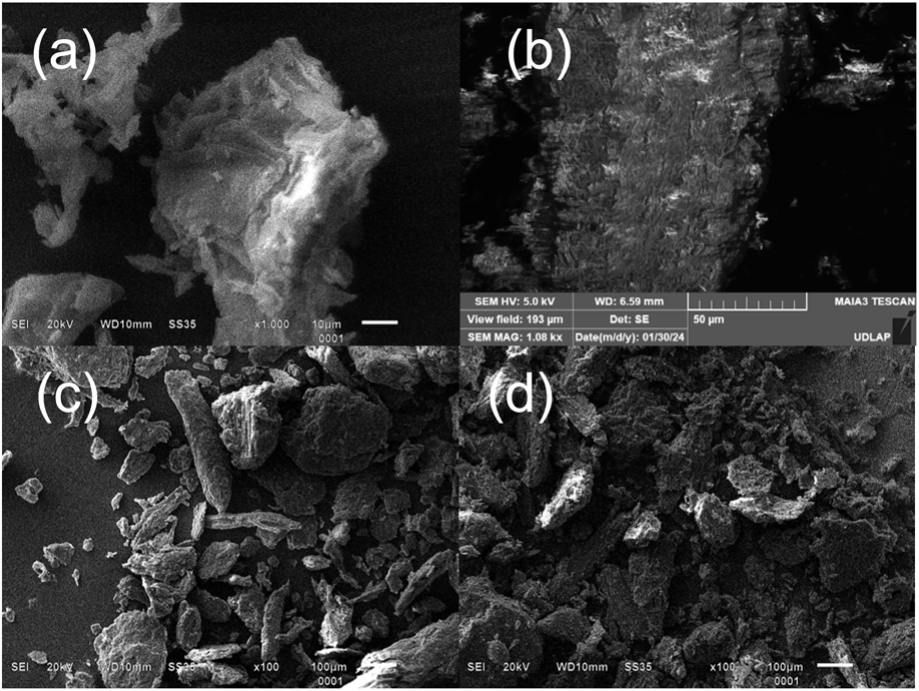
SEM micrographs of (a) CPH, (b) ANCSH0.1, (c) ANCSH5, and (d) ANCSA10. Experimental conditions: accelerating voltage 5–20 kV, magnifications 100×–1000×.

Fig. 3 shows SEM images of CPH, ANCSH0.1, ANCSH5, and ANCSA10 captured at magnifications of 100× and 1000×. The surface morphology of CPH undergoes significant changes following chemical activation. As seen in low-magnification images, CPH initially exhibits a dense, rigid, and compact structure with a smooth surface and no visible cavities. However, after treatment with acid and alkaline agents, the morphology changes dramatically, particularly in samples ANCSH5 and ANCSA10. These chemically activated variants display rougher surfaces and a more porous structure, characterised by the development of additional pores and cracks. This transformation increases the overall surface area of the material, enhancing its potential for applications such as adsorption. The clear contrast between the untreated CPH and the acid/alkaline-treated forms underscores the effectiveness of chemical activation in modifying surface characteristics and improving functional performance.^12,23,24^

The predominantly quasi-spherical and rod morphology of ANCSH5 and ANCSA10 particles is evident, with most seen particles exhibiting this shape. This morphological transformation is primarily attributed to the elimination of hydrophobic waxes, pectin, hemicellulose, lignin, and other impurities from the CPH matrix during sequential acid and alkaline treatments. The defibrillation of cellulose into individual fibrils is linked to the removal of its amorphous regions, predominantly influenced by the presence of hemicellulose and lignin.^18,21,27^ The total weight loss of the CPH, 18% after the NaOH treatment and 40% after the H_2_SO_4_ treatment, further confirms this.

**Fig. 4:**
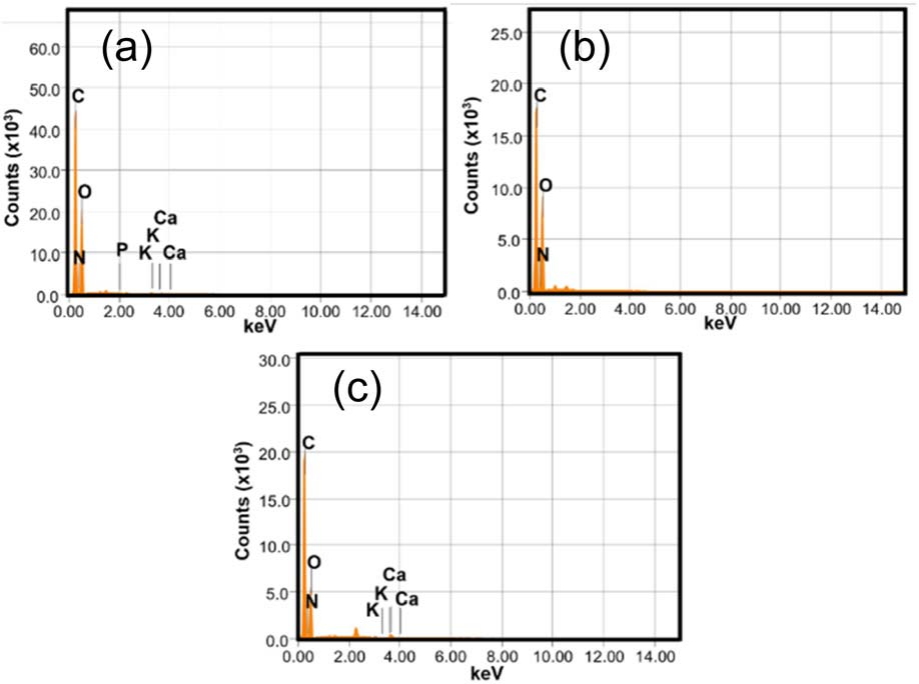
EDX spectra of (a) CPH, (b) ANCSH5, and (c) ANCSA10.

Fig. 4 shows the EDX Spectra of CPH, ANCSH5, and ANCSA10. Elemental analysis of CPH reveals the presence of carbon (47.40%), oxygen (38.32%), nitrogen (13.93%), potassium (0.15%), phosphorus (0.04%), and calcium (0.16%). However, elemental analysis of ANCSH5 and ANCSA10 reveals the presence of all elements detected in CPH, except for phosphorus, potassium, and calcium. The increased carbon peaks (Fig. 4b and c) and higher estimated mass percentages (Table 4) seen in the EDX analysis of ANCSA10 and ANCSH5, relative to CPH, indicate that the chemical treatment effectively promoted the carbonization process.

**Table 4:** EDX semiquantitative analysis of samples: CPH, ANCSA10, and ANCSH5. N.D. not detected

Chemical element (ms%)	CPH	ANCSA10	ANCSH5
C	47.40	49.19	48.91
N	13.93	21.70	20.36
O	38.32	28.8	30.73
K	0.15	0.01	N.D.
P	0.04	N.D.	N.D.
Ca	0.16	0.30	N.D.
Total	100	100	100

Similar outcomes were obtained by Mousavi-Qeydary *et al.*^75^ in their study of activated carbon made from human hair waste, and Putra Negara *et al.*^76^ who synthesised activated carbons derived tabah bamboo.

#### 3.2.2 X-Ray Diffraction (XRD)

**Fig. 5:**
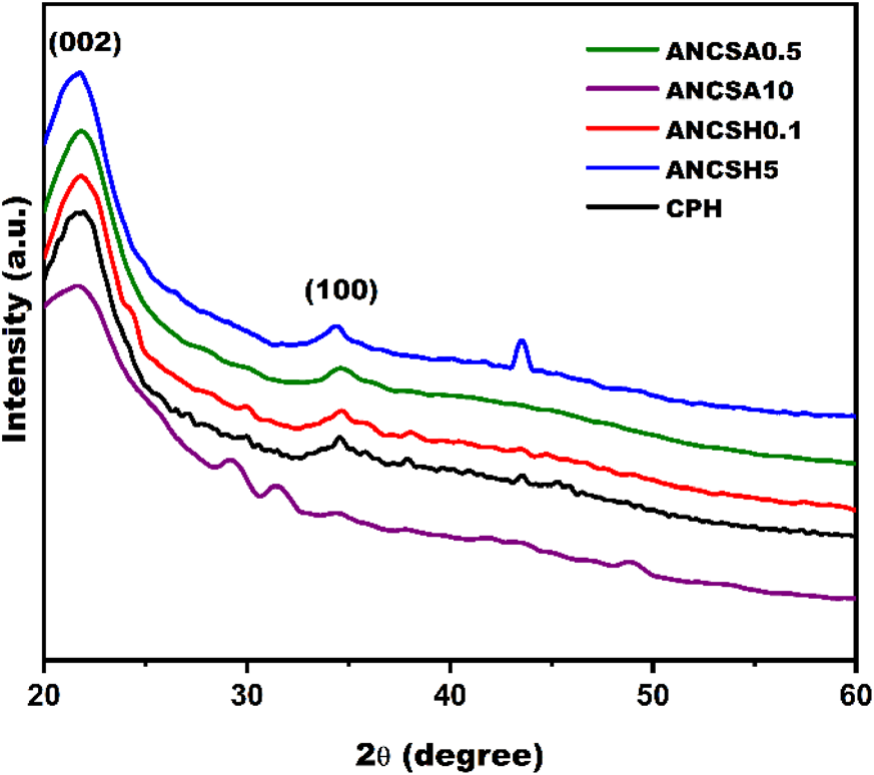
XRD patterns of raw CPH and chemically ANCs: ANCSH0.1, ANCSH5, ANCSA0.5, and ANCSA10.

The XRD pattern of raw lignocellulosic CPH (Fig. 5) exhibited broad, low-intensity peaks at 2*θ* ≈ 21.7°, 34.4°, and 43.5°, corresponding to amorphous cellulose, disordered lignin domains, and the incipient (100) graphitic plane, respectively. These features confirm the highly amorphous nature of the pristine biomass, dominated by poorly ordered carbonaceous structures.^47,49,77^

Significant structural transformations were seen following chemical activation. The ANCSH5 sample (treated with 5 M NaOH) displayed sharper and more intense reflections at the same positions, indicating enhanced crystallinity and a higher degree of graphitization. Notably, the increased intensity of the (100) peak at 43.5° suggests the formation of graphene-like sp^2^ carbon domains, likely due to the effective delignification and removal of hemicellulose under strong alkaline hydrolysis.^48^

In contrast, the ANCSA10 sample (treated with 10 M H_2_SO_4_) showed additional peaks at 29.4° and 31.7°, alongside those at 21.7°, 34.4°, and 43.5°. These are attributed to the formation of organosulfate or sulfonated crystalline phases, reflecting more complex structural rearrangements. However, the broader peaks indicate that strong acid activation induced partial structural collapse, limiting long-range order.^47,52^

Samples treated with lower concentrations of acid (ANCSA0.5) and base (ANCSH0.1) exhibited only weak reflections at 34.4° and 43.5°, indicating limited carbon ordering and incomplete removal of amorphous constituents.

The prominent reflection at 43.5°, corresponding to the (100) plane of hexagonal graphite, serves as a key indicator of aromatic ring condensation and planar sp^2^ carbon alignment.^48,78^ Its pronounced intensity in ANCSH5 confirms the highest degree of graphitisation among all tested materials, consistent with its enhanced physicochemical properties for adsorption applications.

#### 3.2.3 Raman spectroscopy

**Fig. 6:**
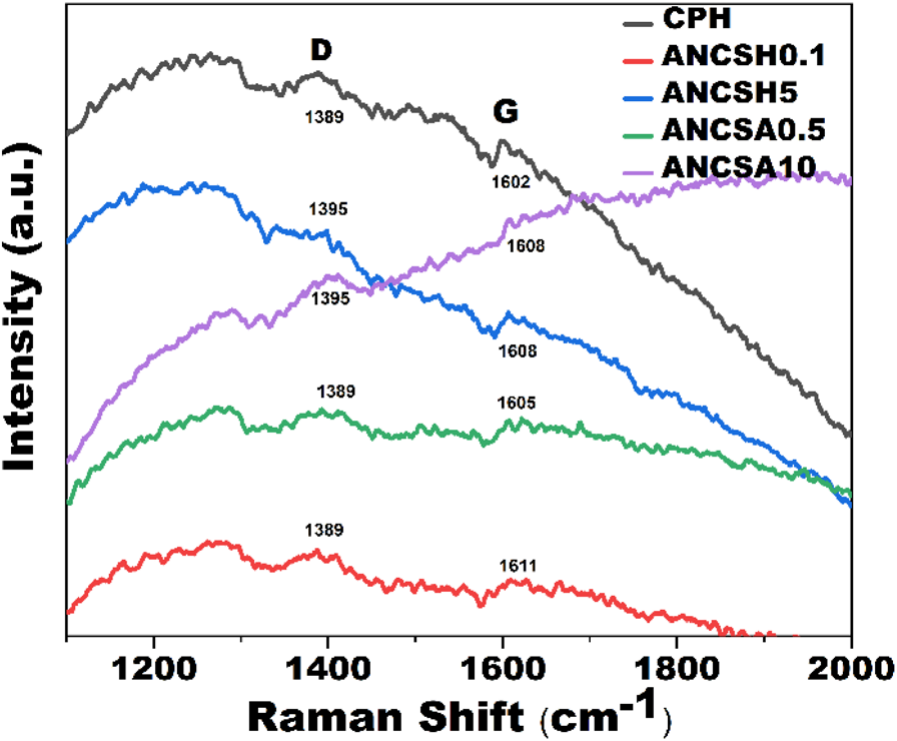
Raman spectra of CPH, ANCSH0.1, ANCSH5, ANCSA0.5, and ANCSA10. Excitation wavelength: 632.8 nm.

Fig. 6 presents the Raman spectra of CPH, ANCSH0.1, ANCSH5, ANCSA0.5, and ANCSA10. The CPH sample exhibited D and G bands at 1389 cm^−1^ and 1602 cm^−1^, respectively. For ANCSH0.1, the D and G bands appeared at 1389 cm^−1^ and 1611 cm^−1^, while for ANCSH5, they were seen at 1395 cm^−1^ and 1608 cm^−1^. In the case of ANCSA0.5, the D and G bands were located at 1389 cm^−1^ and 1605 cm^−1^, respectively. ANCSA10 showed D and G bands at 1395 cm^−1^ and 1608 cm^−1^.

The presence of both D and G bands in all samples confirms the carbonaceous nature of the materials, with varying degrees of structural order. The D and G bands were seen in the ranges of 1300–1410 cm^−1^ and 1550–1610 cm^−1^, respectively, consistent with reported values in the literature for disordered carbon matrices.^50,79,80^ The D band, indicative of structural disorder, exhibited the highest intensity in the raw CPH sample, reflecting a highly disordered carbon framework. In contrast, this behaviour differed in the chemically treated samples, suggesting modifications in the carbon framework due to the treatments.^81–83^ The D band is associated with disordered carbon regions, and the G band corresponds to ordered graphitic structures. The D band arises from the breathing modes of sp^2^-hibridised carbon atoms in aromatic rings and is activated by structural defects or disorder, often associated with sp^3^ hybridisation. In contrast, the G band results from the in-plane stretching of sp^2^-hybridised carbon atoms, characteristic of ordered graphitic domains. These well-defined features in the Raman spectra offer valuable insight into the heterogeneous microstructure of the carbon materials.^79,83,84^

To evaluate the degree of graphitisation, the intensity ratio of the D peak and G peaks was calculated, where:21
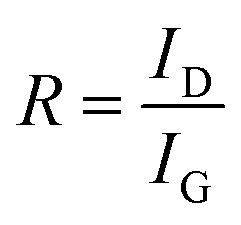
where *R* is the degree of graphitization of carbon materials, *I*_D_ is the intensity of the D peak, *I*_G_ is the intensity of the G peak. This ratio is widely used as a semi-quantitative indicator of structural disorder in carbon materials.^83–85^

In samples treated with sodium hydroxide, particularly ANCSH5, the D band intensity exceeded that of the G band, resulting in higher *R* values. In contrast, sulphuric acid-treated samples did not exhibit the same trend. Interestingly, higher *R* values, often associated with greater *I*_D_, may also reflect an increased presence of aromatic ring structures and a partial reduction in certain types of carbon-related defects.^79,85,86^ This structural evolution can enhance the formation of oxygen-containing functional groups on the material surface, thereby improving adsorption performance by increasing surface polarity and providing additional active sites for interaction with adsorbate molecules such as dyes. Overall, sodium hydroxide treatment promotes a higher degree of graphitisation compared to sulphuric acid treatment of the raw biomass.^41,81^

#### 3.2.4 Fourier Transform Infrared Spectroscopy (FTIR)

**Fig. 7:**
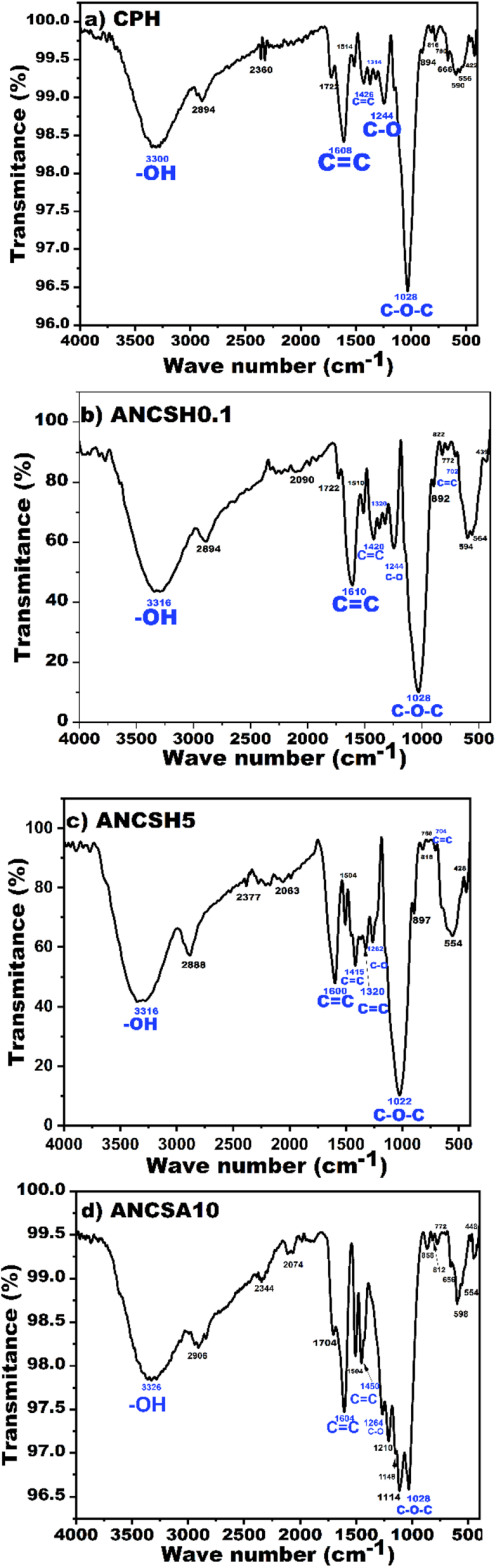
FTIR spectra of (a) CPH, (b) ANCSH0.1, (c) ANCSH5, and (d) ANCSA10.

FTIR was employed to analyse the chemical transformations of functional groups in CPH following chemical activation with sodium hydroxide (NaOH, 0.1 M and 5 M) and sulphuric acid (H_2_SO_4_, 10 M) during the delignification process. As shown in Fig. 7a, the FTIR spectrum of untreated CPH reveals characteristic absorption bands of lignocellulosic biomass. A broad band near ∼3400 cm^−1^ corresponds to O–H stretching vibrations associated with hydrogen bonding or adsorbed moisture, indicative of free hydroxyl groups on the surface.^87^ The absorption at ∼2894 cm^−1^ is attributed to C–H stretching of aliphatic, CH_2_/–CH_3_ groups, linked to cellulose and hemicellulose structures.^24^ The band at 1722 cm^−1^ corresponds to C=O stretching in ester or carboxylic groups, likely originating from hemicellulose or lignin.^20,87^ The signal at ∼1600 cm^−1^ is attributed to aromatic C=C stretching or conjugated C=O groups within lignin, and may also reflect π–π interactions associated with emerging graphitised domains.^5,55,89^ The peak at 1426 cm^−1^ corresponds to aromatic skeletal C=C stretching,^90^ while 1314 cm^−1^ is assigned to in-plane C=C vibrations and symmetric C–H bending of methyl/methylene groups, common in lignin side chains and polysaccharide residues.^88^ A strong band at 1244 cm^−1^ corresponds to C–O–C ether bonds in lignin,^21^ and the absorption at 1028 cm^−1^ is assigned to C–O stretching of primary and secondary alcohols in cellulose and hemicellulose.^5,21^ Finally, the band near 590 cm^−1^ corresponds to out-of-plane C–H bending vibrations.^62^

In contrast, the FTIR spectra of NaOH-treated samples (Fig. 7b and c) show a progressive attenuation and disappearance of the 1722 cm^−1^ band, particularly in the ANCSH5 sample, indicating cleavage of ester linkages and effective removal of hemicellulose.^24^ Similarly, the disappearance of bands at 1244 cm^−1^ and 590 cm^−1^ confirms lignin degradation. This depolymerization process exposes hydroxyl and phenolic groups, thereby enhancing surface reactivity.^23^ The alkaline treatment thus results in significant removal of recalcitrant fractions and increases cellulose accessibility, which is critical for adsorption efficiency.^20^ Notably, new bands appeared at 702 cm^−1^ (ANCSH0.1) and 704 cm^−1^ (ANCSH5), associated with out-of-plane C–H bending in aromatic rings, suggesting the development of π–π stacking interactions between carbon domains, possibly linked to partially graphitised structures.^19,80^

In the case of the acid-treated sample (Fig. 7d), the 1722 cm^−1^ band was red-shifted to 1704 cm^−1^, indicating possible alterations in carbonyl environments due to partial conjugation. While the 1244 cm^−1^ band (associated with either C–O-linkages in lignin) disappeared, suggesting partial lignin removal, the 590 cm^−1^ band remained, indicating incomplete depolymerization of aromatic structures. In addition, intensified bands in the 1210–1114 cm^−1^ region were seen, associated with C–OH and C–O–C stretching, which are indicative of newly formed oxygen-containing acidic functionalities such as lactones, anhydrides, and phenols.^24,55,89^ Notably, unlike the alkali-treated samples (ANCSH0.1 and ANCSH5), which exhibited new bands at 702 and 704 cm^−1^, respectively, attributed to π–π stacking interactions, these bands were absent in the acid-treated ANCSA10. The disappearance of the 1314 cm^−1^ band in ANCSA10 further supports the conclusion that sulphuric acid treatment was less effective for delignification and the development of aromatic carbon structures.

The band at 1028 cm^−1^, attributed to C–O stretching vibrations of primary and secondary alcohols in cellulose and hemicellulose, remained prominent after sulphuric acid treatment (ANCSA10), but showed a notable increase in intensity in the NaOH-treated samples (ANCSH0.1 and ANCSH5). This enhancement suggests a more effective removal of hemicellulose and lignin by the alkaline treatment, resulting in greater exposure of cellulose hydroxyl groups. These newly accessible polar functionalities can contribute significantly to adsorption performance by enabling electrostatic interactions and hydrogen bonding with cationic pollutants. Similar trends have been reported in other studies, where alkaline pretreatment led to a stronger 1028 cm^−1^ signal due to increased cellulose accessibility.^59,91,92^

The more effective delignification achieved through NaOH treatment is attributed to enhanced cellulose exposure and the formation of surface oxygen-containing functional groups such as carbonyl, carboxyl, and hydroxyl groups. These functionalities are known to enhance adsorption performance through mechanisms such as electrostatic attraction, hydrogen bonding, and metal–ligand complexation.^5^ According to previous studies, the nature and abundance of surface functional groups significantly influence the adsorption mechanism, with –COOH and –OH groups promoting the uptake of polar and ionic contaminants, including dyes and heavy metals.^55,89^ Therefore, the applied chemical treatments effectively altered the surface chemistry of CPH, promoting the generation of active sites and improving the material's affinity toward the target contaminant.

Although pure graphitic carbon exhibits minimal infrared activity due to its symmetrical and non-polar structure, partially graphitised domains formed during thermal activation of cellulose produce discernible FTIR bands. These include aromatic C=C stretching near 1600–1610 cm^−1^, for all materials, as well as bands near 1415–1426, 1314–1320, for CPH, ANCSH0.1, and ANCSSH5; and 702–704 cm^−1^. These latter bands are exclusively presented for ANCSH0.1 and ANCSH5, which are associated with sp^2^-hybridised carbon structures and π–π interactions, evidencing the alkaline treatment effect. These features reflect the evolution of oxygenated amorphous carbon networks derived from lignocellulosic precursors. Notably, lignin's inherent aromatic ring structures contribute to π-conjugated domains, facilitating π–π stacking interactions with aromatic pollutants such as cationic methylene blue (MB^+^), which enhances adsorption efficiency, especially in alkaline-treated materials.^24,40,41,89^

FTIR analysis corroborated the structural changes identified by Raman spectroscopy and XRD. The presence of bands near 1600 cm^−1^ (C=C stretching) and 1220–1260 cm^−1^ and 1028 cm^−1^ (C–O stretching) confirms the formation of conjugated aromatic systems and oxygen-rich functionalities during chemical activation. These observations align with the D and G bands in Raman spectra, which signify the coexistence of disordered and graphitic carbon domains. Additionally, the broad peaks in the XRD patterns indicate the presence of amorphous or turbostratic carbon, supporting the formation of partially graphitised structures. Together, these spectroscopic and diffraction analyses demonstrate the successful conversion of CPH into a carbonaceous adsorbent with structural features favorable for π–π interactions and electrostatic adsorption mechanisms.^5,20,41,55^

### 3.3 Adsorption studies

The adsorption performance of MB was evaluated using untreated CPH and chemically modified CPH powders (ANCSA10 and ANCSH5) under identical conditions: 30 °C, pH 7, and an initial dye concentration of 5 mg L^−1^. The removal efficiency followed the order: ANCSH5 > ANCSA10 > CPH. The untreated CPH achieved a removal efficiency of 24% (0.30 mg g^−1^), while ANCSA10 reached 44% (0.50 mg g^−1^). Remarkably, the alkaline-treated sample ANCSH5 exhibited a substantial improvement in MB removal (92%, 1.155 mg g^−1^), highlighting the effectiveness of the NaOH activation in enhancing adsorption capacity.

Beyond adsorption efficiency, the sustainability of ANCSH5 is intrinsically linked to the valorisation of CPH, a locally abundant agro-waste with limited alternative applications. Its conversion into carbon prevents biomass accumulation in landfills, where uncontrolled biological decomposition generates methane (CH_4_), a potent greenhouse gas.^22,38^ In addition, the synthesis employed here requires only sulphuric acid and sodium hydroxide pretreatments at moderate conditions, without the need for high-temperature furnaces, thus minimizing energy consumption and the associated carbon footprint.^39,47^ These attributes distinguish the present route from other biomass-based carbons and further from petroleum-derived ACs, whose production pathways involve fossil feedstocks with significant emissions of GreenHouse Gases (GHGs) and Volatile Organic Compounds (VOCs).^15,77^ This highlights the “green” positioning of the proposed synthesis, which integrates waste reduction, low-energy processing, and circular economy principles.

This improvement may be attributed to the removal of organic compounds during the delignification process facilitated by alkaline treatment, which enhances the chelating ability of the adsorbent surface. Additionally, sodium hydroxide activation promotes a higher degree of graphitization compared to sulphuric acid treatment, leading to the formation of oxygen-containing functional groups. These modifications enhance surface polarity and create additional active sites, thereby enhancing the overall adsorption performance.

#### 3.3.1 Adsorption kinetics

**Fig. 8:**
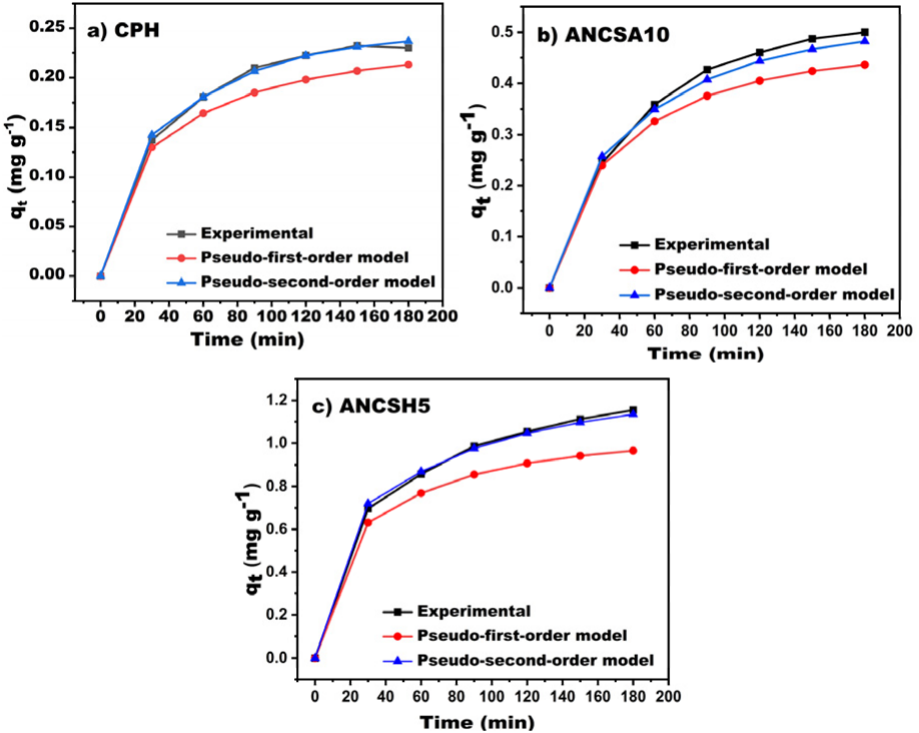
Adsorption kinetics of MB on (a) CPH, (b) ANCSA10, and (c) ANCSH5. Experimental conditions: *C*_0_ = 5 mg L^−1^, pH 6, *T* = 30 °C.

The adsorption kinetics of MB onto CPH, ANCSH5, and ANCSA10 are presented in Fig. 8. A rapid increase in adsorption capacity was seen within the first 60 min for CPH and ANCSH5, indicating high initial affinity towards MB. In contrast, ANCSA10 exhibited a slower uptake, reaching maximum adsorption at 120 min. Equilibrium was established at approximately 120 min for CPH and ANCSH5, while ANCSA10 achieved equilibrium after 150 min. The kinetic data were fitted to pseudo-first-order and pseudo-second-order models (Table 5). The pseudo-second-order model provided superior *R*^2^ and closer agreement between experimental and calculated adsorption capacities (*q*_*e*_, exp and *q*_*e*_, cal), indicating its better suitability for describing the system. These results suggest that the adsorption mechanism is governed predominantly by chemisorption processes, potentially involving electron sharing or exchange, such as surface complexation, ion exchange, or precipitation.^41,93,94^

Kinetic parameters of MB on CPH, ANCSH5, and ANCSA10 obtained from the pseudo-first-order and pseudo-second-order models

**Table:** 

Adsorbent	Pseudo-first-order kinetics		
	*k*_1_ (min^−1^)	*q*_*e*_ (mg g^−1^)	*R* ^2^
CPH	0.02513	0.21966	0.99826
ANCSH5	0.03045	0.71546	0.96173
ANCSA10	0.02825	0.99607	0.65892

**Table:** 

Adsorbent	Pseudo-second-order kinetics		
	*k*_2_ (g mg^−1^ min^−1^)	*q*_*e*_ (mg g^−1^)	*R* ^2^
CPH	0.12188	0.27490	0.99827
ANCSH5	0.11569	1.18085	0.99918
ANCSA10	0.00005	7.57070	0.98107

#### 3.3.2 Isotherm experiments

The nature of the adsorption process was determined using different adsorption isotherm models to establish the best-fitted isotherm. The adsorption equilibrium data for MB on the three adsorbents (CPH, ANCSA10, and ANCSH5) were fitted to the Langmuir and the Freundlich models. The resulting parameters and *R*^2^ are summarised in Table 6.

Calculated equilibrium constants of MB on CPH, ANCSH5, and ANCSA10 obtained from the Langmuir and the Freundlich isotherm models

**Table:** 

Adsorbent	Langmuir isotherm		
	*q*_max_ (mg g^−1^)	*K*_L_ (L mg^−1^)	*R* ^2^
CPH	100.0000	0.002418	0.5291
ANCSH5	100.0000	0.024424	0.9997
ANCSA10	100.0000	0.007475	0.1416

**Table:** 

Adsorbent	Freundlich isotherm		
	*K*_F_ (mg^1−1/*n*^ g^−1^ L^1/*n*^)	*n*	*R* ^2^
CPH	0.000016	0.201033	0.8449
ANCSH5	2.377979	1.037309	0.9999
ANCSA10	0.356150	0.687208	0.1559

The Langmuir model, which assumes monolayer adsorption onto a homogeneous surface with finite, identical sites, showed a strong correlation for ANCSH5 (*R*^2^ = 0.9997). This suggests that the adsorption onto ANCSH5 may involve more uniform active sites with higher energy affinity toward MB. In contrast, CPH and ANCSA10 yielded low Langmuir *R*^2^ values (0.53 and 0.14, respectively), indicating that the assumption of surface homogeneity does not hold for these materials.

The Freundlich model, which accounts for adsorption on heterogeneous surfaces, provided a better fit for CPH (*R*^2^ = 0.85) and ANCSH5 (*R*^2^ = 0.9999), while it still performed poorly for ANCSA10 (*R*^2^ = 0.16). These differences may be attributed to variations in surface functionalization and porosity induced by chemical treatment, as supported by FTIR and BET analyses.

**Fig. 9:**
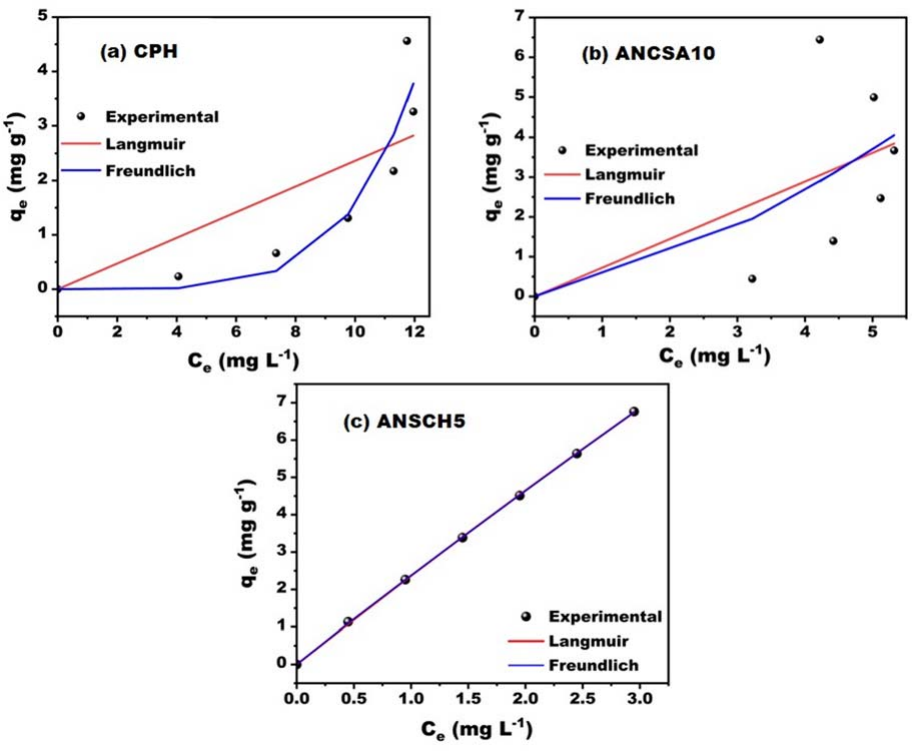
Experimental adsorption isotherms (*q*_*e*_*vs. C*_*e*_) of MB on (a) CPH, (b) ANCSA10, and (c) ANCSH5, fitted with Langmuir and Freundlich models.

As shown in Fig. 9, the experimental adsorption data were fitted using the non-linear forms of the Langmuir and the Freundlich isotherm models. The results indicate an excellent fit for ANCSH5, moderate agreement for CPH, and poor conformity for ANCSA10, highlighting differences in surface characteristics and adsorption mechanisms among the adsorbents. In particular, CPH and ANCSA10 exhibited non-ideal and progressive adsorption behaviour. These deviations can be attributed to surface heterogeneity, low adsorbate–adsorbent affinity, and multilayer adsorption factors not fully captured by the monolayer assumption of the Langmuir model or the empirical approximation inherent to the Freundlich model. Importantly, although the equilibrium data showed deviations from classical isotherms, the adsorption process itself was effective, as evidenced by the high MB removal percentages and the excellent agreement of the kinetic data with the pseudo-second-order model. This reinforces the idea that adsorption performance should not be evaluated solely based on isotherm conformity, but also considering the dynamic interaction mechanisms and material-specific properties.

**Fig. 10:**
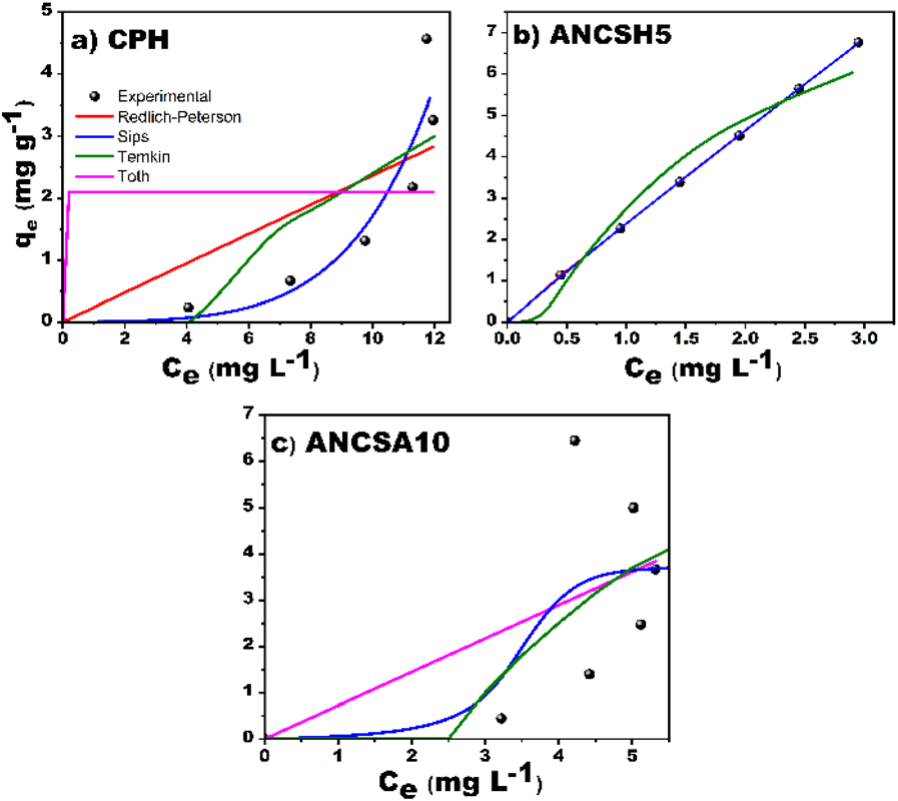
Experimental adsorption isotherms (*q*_*e*_*vs. C*_*e*_) of MB on (a) CPH, (b) ANCSH5, and (c) ANCSA10, fitted with extended isotherm models (Redlich–Peterson, Sips, Temkin, and Toth).

To further explore the equilibrium behaviour of MB adsorption onto the three adsorbents, extended isotherm models, including the Sips, Redlich–Peterson, Temkin, and Toth models, were applied. These models are useful for describing adsorption on heterogeneous surfaces, mixed adsorption mechanisms, or multilayer phenomena not captured by classical models. Fig. 10 shows the plots for the extended isotherm models (Redlich–Peterson, Sips, Temkin, and Toth), and the calculated isotherm constants are summarised in Table 7.

Calculated equilibrium constants of MB on CPH, ANCSH5, and ANCSA10 obtained from the Sips, Redlich–Peterson, Temkin, and Toth models

**Table:** 

Adsorbent	Sips isotherm			
	*q*_max_ (mg g^−1^)	*K*_s_ (L mg^−1^)	*n*	*R* ^2^
CPH	100.0000	0.0443	5.0955	0.8444
ANCSH5	100.0000	0.0248	1.0053	0.9997
ANCSA10	3.9212	0.2869	10.0000	0.2948

**Table:** 

Adsorbent	Redlich–Peterson isotherm			
	*K*_R_ (L g^−1^)	*a*_R_	*g*	*R* ^2^
CPH	18.0386	75.3073	0.0000	0.5357
ANCSH5	100.0000	41.0527	0.0368	0.9999
ANCSA10	42.2199	57.4525	0.0000	0.1431

**Table:** 

Adsorbent	Toth isotherm			
	*q*_max_ (mg g^−1^)	*K*_T_ (L mg^−1^)	*t*	*R* ^2^
CPH	2.0345	14.4479	23.4162	0.0000
ANCSH5	100.0000	0.0241	1.0793	0.9998
ANCSA10	13.7814	0.0524	60.5573	0.1431

**Table:** 

Adsorbent	Temkin isotherm		
	*A* (L g^−1^)	*B*	*R* ^2^
CPH	0.2207	3.0693	0.6126
ANCSH5	2.6914	2.9228	0.9306
ANCSA10	0.4074	5.3564	0.1959

The Sips model, which combines elements of Langmuir and Freundlich, yielded excellent correlation for ANCSH5 (*R*^2^ = 0.9997), consistent with its previously seen near-monolayer behaviour with some degree of heterogeneity. For CPH, the model improved the fit moderately (*R*^2^ = 0.84), suggesting a surface with variable site energies. In contrast, ANCSA10 showed a limited correlation (*R*^2^ = 0.29), indicating that the model assumptions may not apply to its surface characteristics.

The Redlich–Peterson model, capable of bridging both Langmuir and Freundlich forms, also provided an exceptional fit for ANCSH5 (*R*^2^ = 0.9999). Its performance was slightly lower for CPH and poor for ANCSA10, again reflecting heterogeneity and non-ideal adsorption patterns. The Temkin model, which assumes a linear decrease in adsorption energy with coverage, showed moderate fits for CPH and ANCSH5, while underperforming for ANCSA10. This suggests that adsorbent–adsorbate interactions in these systems involve energetic heterogeneity or weak bonding effects. The Toth model, often used for highly heterogeneous systems, resulted in the best fit for ANCSH5 (*R*^2^ ≈ 1) and improved fit for CPH compared to classical models. However, ANCSA10 again showed poor adjustment, highlighting its distinct and possibly multilayer or diffusion-limited adsorption mechanism.

ANCSH5 exhibited strong agreement with all models, particularly Redlich–Peterson and Toth, indicating a highly efficient and energetically accessible surface. The similarity in performance among classical and extended models for ANCSH5 suggests well-distributed active sites, moderate surface heterogeneity, and favorable interaction with the adsorbate. In contrast, ANCSA10 showed poor correlation across all models, indicating deviation from ideal or energetically uniform adsorption behaviour likely due to severe surface heterogeneity, inaccessible sites, or multilayer interactions. CPH showed moderate fits, with Sips and Temkin performing better.

In summary, the application of extended isotherm models supports the conclusion that ANCSH5 possesses a surface with high affinity and partially homogeneous active sites, whereas CPH shows heterogeneous behaviour with moderate adsorption energy. The poor fit of all models to ANCSA10 underscores the need for either customised models or hybrid approaches for complex surfaces that incorporate kinetic or diffusional constraints.

##### 3.3.2.1 Model comparison based on coefficient of determination

**Fig. 11:**
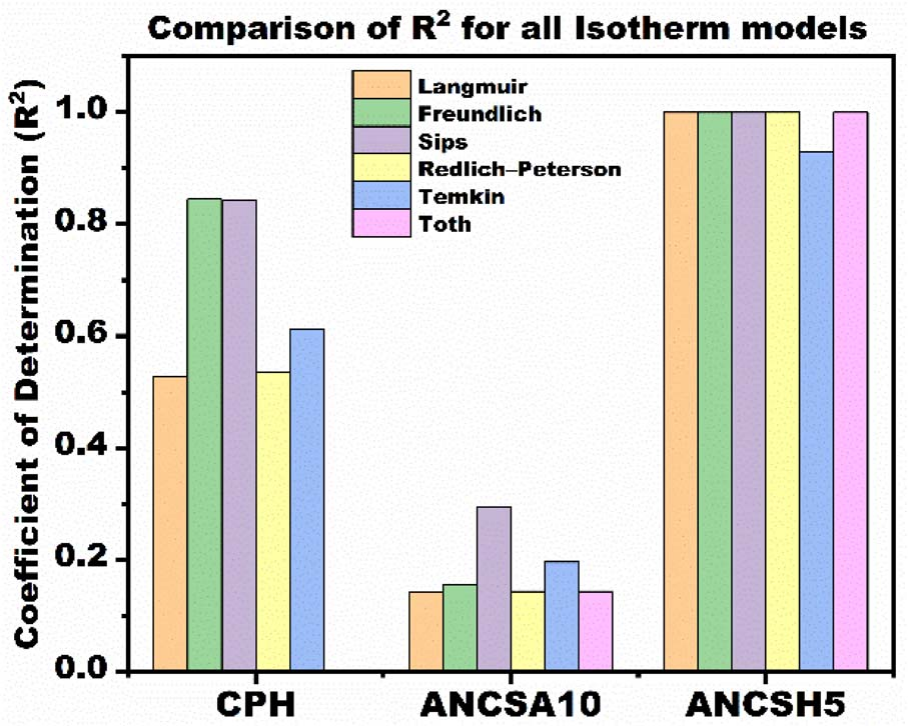
Comparison of *R*^2^ obtained from classical and extended isotherm models for MB adsorption onto CPH, ANCSA10, and ANCSH5.

To systematically compare the performance of classical and extended isotherm models, *R*^2^ was calculated for each model and adsorbent (Table 8). This comparison reveals substantial differences in model performance depending on the material's surface chemistry and adsorption behaviour (Fig. 11).

**Table 8:** *R* ^2^ for all isotherm models

Adsorbent	Langmuir	Freundlich	Sips	Redlich–Peterson	Temkin	Toth
CPH	0.5291	0.8449	0.8444	0.5357	0.6126	0.0000
ANCSA10	0.1416	0.1559	0.2948	0.1431	0.1959	0.1431
ANCSH5	0.9997	0.9999	0.9997	0.9999	0.9306	0.9998

Among the three adsorbents, ANCSH5 consistently exhibited the highest *R*^2^ values across all models, with nearly perfect fits for the Sips, Toth, and Redlich–Peterson models (*R*^2^ > 0.999). This indicates a well-defined and partially homogeneous adsorption mechanism, likely involving both high surface area and active functional groups enhanced by NaOH activation.

In contrast, CPH showed moderate fits, with the Freundlich and Sips models providing the best results (*R*^2^ ≈ 0.84). The relatively low *R*^2^ values for Langmuir and Redlich–Peterson suggest that adsorption onto CPH occurs on a heterogeneous surface without saturation, likely due to limited availability or diversity of active sites.

ANCSA10 exhibited the lowest *R*^2^ values across nearly all models, including extended ones. This suggests a highly complex or non-ideal adsorption process, potentially involving multilayer formation, diffusion limitations, or weak interactions.

These findings reinforce the importance of applying a range of isotherm models, classical and extended, when characterizing novel adsorbents. Relying solely on Langmuir or Freundlich models may obscure underlying mechanistic differences, particularly in bio-based or structurally diverse materials, and reveal significant differences in adsorption capacity and surface affinity among the adsorbent materials.

#### 3.3.3 Thermodynamic studies

Thermodynamic parameters were evaluated using the van't Hoff approach based on equilibrium constants (*K*_c_ = *q*_*e*_/*C*_*e*_) obtained from isotherm data at different temperatures (25–45 °C). The standard Gibbs free energy (Δ*G*°), enthalpy (Δ*H*°), and entropy (Δ*S*°) were calculated according to eqn (22–24):

22 Δ*G*^0^ = −*RT* ln *K*_C_

23
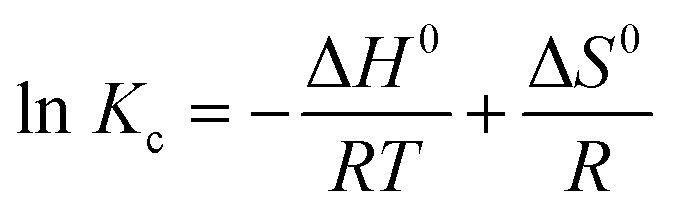

24 Δ*G*^0^ = Δ*H*^0^ − *T*Δ*S*^0^

Table 9 summarises the calculated thermodynamic parameters. Negative values of Δ*G*° across all tested temperatures confirm the spontaneous nature of MB adsorption onto ANCSH5, consistent with observations reported for other biomass-derived adsorbents.^9,57,95^ The positive Δ*H*° (25.0 kJ mol^−1^) indicates an endothermic process, while positive Δ*S*° values reflect increased randomness at the solid–liquid interface, in agreement with previous studies on dye adsorption involving natural adsorbents.^64^ The magnitude of Δ*H*° suggests that the adsorption involves strong physicochemical interactions, consistent with electrostatic attraction, hydrogen bonding, and π–π stacking mechanisms previously identified. These results corroborate the kinetic and isotherm analyses, demonstrating that MB adsorption on ANCSH5 is thermodynamically favorable, spontaneous, and enhanced at higher temperatures.^9,57,95^

**Table 9:** Thermodynamic parameters for MB adsorption on ANCSH5

*T* (°C)	*T* (K)	Δ*G*° (kJ mol^−1^)	Δ*H*° (kJ mol^−1^)	Δ*S*° (J mol^−1^ K^−1^)
25	298.15	−4.76	25.0	99.8
35	308.15	−5.52	25.0	99.6
45	318.15	−6.28	25.0	99.1

#### 3.3.4 Isotherm modelling and effect of pH on adsorption behaviour

The adsorption performance of CPH, ANCSH5, and ANCSA10 was evaluated through non-linear regression of classical (Langmuir and Freundlich) and extended (Redlich–Peterson, Sips, Toth, Temkin) isotherm models. Isotherm shapes, model parameters, and *R*^2^ revealed significant material-specific differences in adsorption mechanisms.

All experiments were conducted at pH 6, a condition near the slightly acidic regime, which can influence surface charge and adsorbate speciation. At this pH, functional groups such as hydroxyls and carboxyls on the adsorbent surface may be partially deprotonated, enhancing electrostatic attraction with cationic species or promoting hydrogen bonding. This effect is particularly favorable in materials with higher surface polarity and accessible acidic groups.

Among all tested adsorbents, ANCSH5 demonstrated the highest performance, with excellent agreement across all isotherm models (*R*^2^ > 0.99). The strong affinity seen at pH 6 indicates a surface rich in accessible functional groups whose ionization state promotes adsorption through electrostatic interactions and hydrogen bonding. These interactions are likely supported by the mildly acidic conditions, which optimise the charge distribution and polarity of the adsorbate–adsorbent system. This behaviour aligns with reports on functionalised bio-carbons, where pH-induced deprotonation increases the uptake of cationic dyes.^96^

CPH, while exhibiting moderate adsorption capacity, was best described by the Freundlich model (*R*^2^ = 0.788), suggesting adsorption on a heterogeneous surface with sites of varying energy. The lower *R*^2^ values obtained for the Langmuir and extended models indicate that adsorption at pH 6 deviates from ideal monolayer behaviour, potentially due to partial ionization of surface groups or weak adsorbate–adsorbent interactions under mildly acidic conditions. This limited affinity aligns with prior studies on lignocellulosic adsorbents in similar pH environments.^97,98^

In contrast, ANCSA10 showed poor correlation with all isotherm models, including Sips and Toth (*R*^2^ < 0.4), indicating low adsorption capacity and inadequate model description at pH 6. This behaviour may result from surface deactivation, a low density of functional groups, or steric hindrance. The lack of improvement under acidic conditions suggests that its active sites are unresponsive to moderate pH shifts or that other mechanisms, such as deep microporosity, intraparticle diffusion, or non-electrostatic interactions, may dominate. Such deviations from equilibrium-based models have been previously reported for bio-based adsorbents with complex surface architectures.^99^

These findings highlight the crucial role of solution pH in regulating adsorption efficiency, particularly for bio-based adsorbents with heterogeneous surface chemistries. Under uniform pH 6 conditions, ANCSH5 exhibited superior performance, attributed to its surface functionality being well-aligned with mildly acidic environments. In contrast, CPH displayed a moderate adsorption response, while ANCSA10 remained largely unresponsive.

##### 3.3.4.1 Surface charge properties and pH_PZC_ determination

**Fig. 12:**
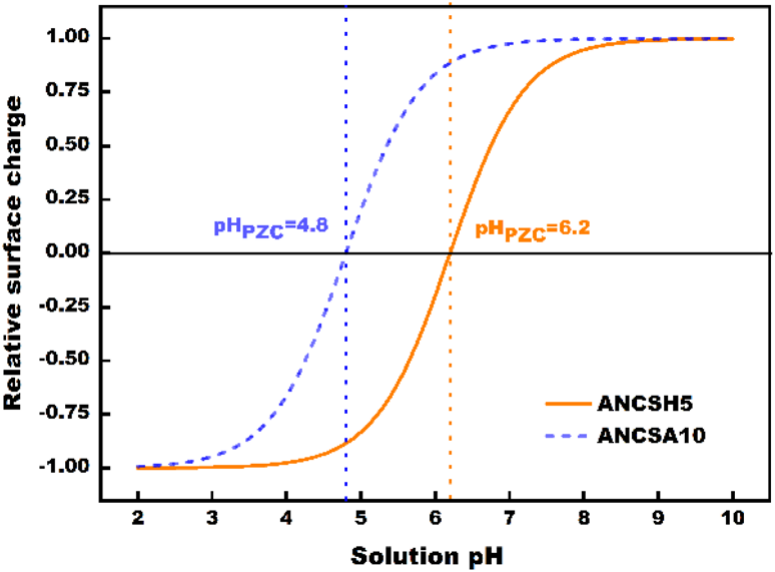
Determination of the pH_PZC_ of ANCSH5 and ANCSA10 using the pH-drift method.

The surface charge characteristics of the adsorbents were evaluated through the point of zero charge (pH_PZC_) determined by the pH-drift method, yielding values of 6.2 for ANCSH5 and 4.8 for ANCSA10 (Fig. 12). These differences have important implications for adsorption behaviour, as the pH_PZC_ defines the pH at which the surface switches from positive to negative charge. When the solution pH exceeds the pH_PZC,_ the surface becomes predominantly negatively charged, enhancing electrostatic attraction towards cationic dyes such as MB^+^; conversely, at pH values below the pH_PZC_, electrostatic repulsion dominates.^38,52^ Similar pH-dependent trends have been reported for biomass-derived carbons and low-cost adsorbents used for cationic dye removal.^12,51,54,55^

The acid dissociation constant (p*K*_a_) of MB is approximately 3.8, indicating that above this pH, the dye predominantly exists in its cationic form (MB^+^) in aqueous solution.^100–102^ Consequently, under the experimental conditions employed in this work (pH 6–7), MB molecules are positively charged and readily interact with negatively charged adsorbent surfaces. When the solution pH is equal to or higher than the pH_PZC_ (6.2 for ANCSH5 and 4.8 for ANCSA10), deprotonation of surface –COOH and –OH groups (–COOH → –COO^−^; –OH → –O^−^) generates a net negative charge, promoting strong electrostatic attraction with MB^+^, in addition to hydrogen bonding and π–π stacking with aromatic domains on the carbon surface.

At near-neutral pH, ANCSH5 therefore exhibits superior adsorption capacity, consistent with its higher pH_PZC_ and greater availability of deprotonated oxygenated groups. Within the working pH window (6–7), the surface of ANCSH5 is partially deprotonated, exposing –COO^−^ and –O^−^ sites that enhance electrostatic interactions with MB^+^, synergistically reinforced by π–π stacking between the dye's aromatic rings and the partially graphitised domains of the adsorbent.^38,52,55^ In contrast, ANCSA10, with a lower pH_PZC_, remains closer to a neutral or slightly positive surface state, limiting the availability of negatively charged sites and thus reducing electrostatic affinity for MB^+^.^12,26,51,54^ This interplay between the dye's p*K*_a_ and the adsorbent's pH_PZC_ is a decisive parameter governing the charge-controlled adsorption mechanism and explains the high adsorption efficiency observed at near-neutral pH.

Table 10 compares the maximum adsorption capacities of ACs derived from various lignocellulosic biomasses for MB removal, predicted by isotherm modelling. Although some biomass-based carbons report higher *q*_max_ values (*e.g.*, coffee husk, pineapple residues), these capacities were typically obtained under extreme conditions, such as very high initial MB concentrations (≥400 mg L^−1^) or excessive adsorbent dosages. By contrast, the NaOH-activated CPH (ANCSH5) synthesised in this work achieved a competitive *q*_max_ of 100 mg g^−1^ under more realistic operating conditions (*C*_0_ = 5–30 mg L^−1^, pH 6, 35 °C, dosage = 4 g L^−1^). This result is particularly relevant for practical wastewater treatment, where pollutant concentrations are moderate and the use of large adsorbent doses is economically unfeasible. Moreover, the integration of statistical optimisation *via* CCD-RSM adds a further competitive edge by ensuring that adsorption performance is guided by validated optimal conditions. The distinctive advantage of ANCSH5 compared with other biomass-derived carbons lies in the unique lignocellulosic composition of CPH, which is notably rich in hemicellulose. This facilitates extensive delignification during alkaline treatment and promotes the development of partially graphitised domains and a high density of oxygen-containing functional groups, as confirmed by Raman, XRD, and FTIR analyses. Unlike conventional precursors such as rice husk or coconut shell, where performance is largely governed by surface area, CPH-derived ANC combines enhanced graphitization with tailored surface chemistry. This synergy provides abundant active sites for electrostatic interactions, hydrogen bonding, and π–π stacking with MB molecules. Collectively, these features distinguish ANCSH5 as a competitive, sustainable, and industrially relevant adsorbent within the broader family of biomass-based carbons.

**Table 10:** Comparative *q*_max_ of biomass-derived carbons for MB removal, obtained from isotherm models^a^

Biomass precursor	Activation/treatment	*q*_max_ (mg g^−1^)	Conditions	Ref.
Coffe husk	Pyrolysis	416.68	MB *C*_0_ range: 50–500 mg L^−1^; pH: 7; *T*: 30 °C; adsorbent dosage: 1 g L^−1^	5
Pineapple leaf powder	Untreated	281	MB *C*_0_ range: 1.6–6.40 mg L^−1^; pH: 7.5; *T*: 24 °C; adsorbent dosage: 0.3 g L^−1^	51
Pineapple waste (leaves, stem, crown)	Pyrolysis	288.34	MB *C*_0_ range: 5–400 mg L^−1^; pH: 7; *T*: 30 °C; adsorbent dosage: 10 g L^−1^	11
Watermelon rings	H_2_SO_4_	200	MB *C*_0_ range: 50–400 mg L^−1^; pH: 5.6; *T*: 30 °C; adsorbent dosage: 0.8 g L^−1^	55
Sugarcane bagasse	Pyrolysis	113.01	MB *C*_0_ range: 20–50 mg L^−1^; pH: 7.4; *T*: 30 °C; adsorbent dosage: 0.6 g L^−1^	3
Banana stem	Pyrolysis	101	MB *C*_0_ range: 25–200 mg L^−1^; pH: 7; *T*: 30 °C; adsorbent dosage: 3 g L^−1^	54
Cocoa pod husk	NaOH	100	MB *C*_0_ range: 5–30 mg L^−1^; pH: 6; *T*: 35 °C; adsorbent dosage: 4 g L^−1^	This study
Yellow passion-fruit peel	Untreated	44.7	MB *C*_0_ range: 5–600 mg L^−1^; pH: 8; *T*: 25 °C; adsorbent dosage: 10 g L^−1^	57
Annona squmosa seed	H_2_SO_4_	25.91	MB *C*_0_ range: 25–200 mg L^−1^; pH: 6; *T*: 27 °C; adsorbent dosage: 4 g L^−1^	96
Brazil nut shell	Untreated	7.81	MB *C*_0_ range: 100–1500 mg L^−1^; pH: 6.5; *T*: 30 °C; adsorbent dosage: 100 g L^−1^	99

a MB C_0_: MB initial concentration range.

##### 3.3.4.2 Surface functionalization and interaction with MB^+^

The chemical activation processes applied to the adsorbents led to the incorporation of oxygen-containing functional groups on the carbon surface. These groups include carboxyl (–COOH), hydroxyl (–OH), and carbonyl (–C=O) moieties, which are commonly formed through oxidation reactions during treatment with either acidic or alkaline agents. At pH 6, carboxylic groups tend to deprotonate, resulting in negatively charged carboxylate (–COO^−^) species, whereas hydroxyl and carbonyl groups contribute to surface polarity and hydrogen bonding capacity. Together, these functionalities enhance the overall hydrophilicity and facilitate electrostatic attraction with the MB^+^ molecules. This explains the improved adsorption performance seen in the NaOH-treated ANCSH5, in contrast to the H_2_SO_4_-activated ANCSA10, which likely retains a more protonated and less negatively charged surface under the same conditions.

FTIR analysis revealed that the band at 1028 cm^−1^, assigned to the C–O stretching of primary and secondary alcohols, became more intense following NaOH treatment, indicating a higher density of exposed hydroxyl groups on the surface. These –OH groups, primarily from cellulose, play a critical role in the adsorption mechanism by participating in hydrogen bonding and electrostatic interactions with the MB^+^ molecules. This is particularly relevant at pH 6, where partial deprotonation of hydroxyl and carboxyl groups enhances surface polarity and negative charge. The increased exposure of C–OH groups, especially in ANCSH5, correlates with the improved adsorption performance seen in kinetic and isotherm studies. Similar findings have been reported in the literature, where stronger C–O bands at ∼1028 cm^−1^ were associated with improved adsorption properties due to enhanced accessibility of cellulose chains after alkaline pretreatment.^59,91,92^

At pH 6, π–π stacking interactions are particularly relevant for the adsorption of MB^+^, given its planar aromatic structure.^24,41,55,89^ The FTIR spectra revealed stronger aromatic C=C bands for ANCSH5, indicating a higher density of conjugated π-systems capable of forming stable interactions with MB^+^. These π–π interactions provide an additional adsorption mechanism beyond electrostatic attraction, especially in cases where the surface charge is neutral or weakly positive (as in ANCSA10). In ANCSH5, both π–π stacking and electrostatic forces act synergistically, leading to enhanced adsorption capacity and model fitting. Furthermore, this pH was selected to simulate mildly acidic wastewater conditions and to promote adsorption *via* electrostatic attraction, hydrogen bonding, and π–π stacking.

**Fig. 13:**
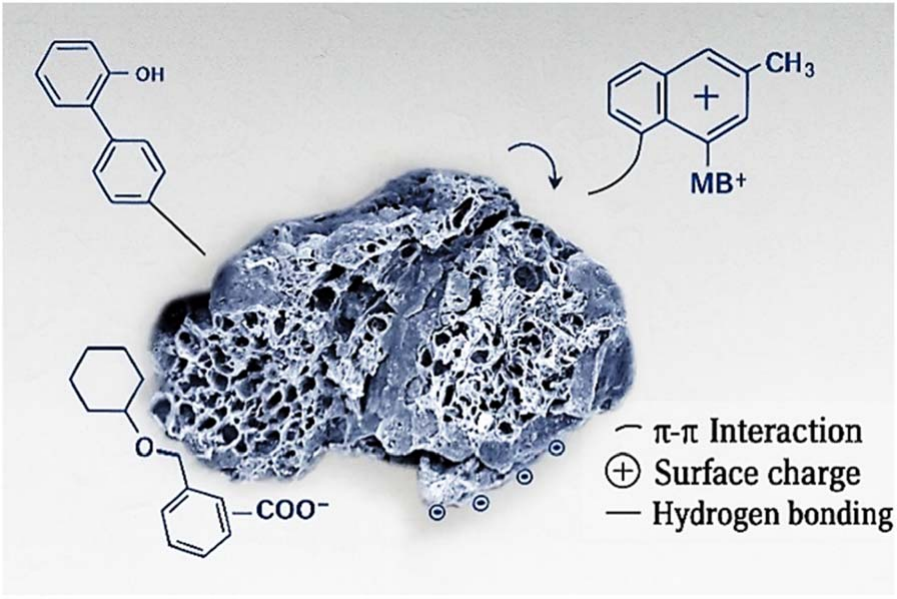
Proposed adsorption mechanism of MB^+^ onto oxygen-functionalised carbon-based adsorbents at pH 6.

Fig. 13 illustrates three primary interactions:

(1) π–π stacking, involving the aromatic rings of MB^+^ and conjugated π-domains on the adsorbent surface.

(2) Electrostatic attraction, driven by the deprotonation of carboxylic groups (–COOH → –COO^−^) on the adsorbent at pH 6, which interacts with the positively charged MB^+^ molecules.

(3) Hydrogen bonding, between surface hydroxyl (–OH) or carbonyl (–C=O) groups and electronegative atoms of MB^+^.

These combined interactions explain the strong affinity of MB^+^ toward materials like CPH and ANCSH5, where both surface polarity and negative charge enhance adsorption efficiency.

#### 3.3.5 Experimental design CCD and optimisation studies

Based on the CCD, the relationship between the process variables (temperature and pH) and the output responses, % MB removal and adsorption capacity, was modeled using a second-order polynomial equation. Table 11 presents both the experimental and predicted adsorption capacities derived from the design matrix. The incorporation of quadratic terms significantly enhanced the model's predictive capability, offering improved flexibility in identifying optimal operating conditions compared to the simpler linear interaction model. According to the RSM results, the following quadratic expression was derived.

25 % Removal = 9.254 + 2.203*T* − 1.596 pH + 0.191*T* pH − 0.0338*T*^2^ − 0.2052 pH^2^

26 % *q* = 0.0900 + 0.0283*T* + 0.0239 pH + 0.0023*T* pH−0.0004*T*^2^ − 0.0028 pH^2^

**Table 11:** Experimental and predicted responses (% removal and adsorption capacity, *q*) obtained from the CCD matrix

Parameters			Response			
			(% Removal, *Y*_1_)		(Adsorption capacity (*q*), mg g^−1^, *Y*_2_)	
Runs order	Temperature, *X*_1_ (°C)	pH, *X*_2_	Experimental	Predicted	Experimental	Predicted
1	35	2	59.51	60.70	0.74	0.76
2	35	6	90.70	87.23	1.13	1.09
3	65	2	35.83	36.88	0.45	0.46
4	65	6	89.92	86.31	1.12	1.07
5	29	4	68.82	69.96	0.86	0.87
6	71	4	51.32	52.65	0.64	0.66
7	50	1	47.90	45.87	0.60	0.57
8	50	7	98.66	100	1.23	1.28
9	50	4	75.13	76.20	0.94	0.95
10	50	4	78.12	76.20	0.98	0.95
11	50	4	76.14	76.20	0.95	0.95
12	50	4	75.20	76.20	0.94	0.95

Tables 12 and 13 summarise the ANOVA results for the quadratic response surface models. The ANOVA for the sorption of MB onto ANCSH5 confirmed the overall statistical significance of the models, with *F*-values of 20.36 for % Removal and 20.69 for adsorption capacity (*q*, mg g^−1^). The corresponding *p*-values (<0.0001) indicate that both regression models are highly significant. Moreover, the *R*^2^, adjusted *R*^2^, and predicted *R*^2^ values for both models exceeded 0.97, demonstrating excellent model fit, generalizability, and predictive reliability.

**Table 12:** ANOVA for the % removal response surface quadratic model in the adsorption of MB by ANCSH5^a^

Source	Sum of squares	d*f*	Mean square	*F*-value	*p*-value
Model	985.48	5	197.10	20.36	0.0011
*X*_1_-temperature	268.62	1	268.62	27.75	0.0019
*X*_2_-pH	226.05	1	226.05	23.35	0.0029
*X*_1_ *X*_2_	131.10	1	131.10	13.54	0.0103
*X*_1_^2^	354.52	1	354.52	36.62	0.0009
*X*_2_^2^	5.19	1	5.19	0.54	0.4916
Residual	58.08	6	9.68		

a *R* ^2^ = 0.9852, adjusted *R*^2^ = 0.9728, predicted *R*^2^ = 0.9852; *p* < 0.05 indicates the model terms are significant; *P* < 0.01 indicates the model terms are highly significant.

**Table 13:** ANOVA for *q* response surface model in the adsorption of MB by ANCSH5^a^

Source	Sum of squares	d*f*	Mean square	*F*-value	*p*-value
Model	0.16	5	0.03	20.69	0.0010
*X*_1_-temperature	0.04	1	0.04	28.57	0.0018
*X*_2_-pH	0.04	1	0.04	23.78	0.0028
*X*_1_ *X*_2_	0.02	1	0.02	12.95	0.0114
*X*_1_^2^	0.06	1	0.06	37.51	0.0009
*X*_2_^2^	0.00	1	0.00	0.65	0.4512
Residual	0.01	6	0.00		

a *R* ^2^ = 0.9850, adjusted *R*^2^ = 0.9725, predicted *R*^2^ = 0.9850; *p* < 0.05 indicates the model terms are significant; *p* < 0.01 indicates the model terms are highly significant.

For % removal, the statistically significant model terms were *X*_1_ (temperature), *X*_2_ (pH), *X*_1_^2^ (temperature^2^), and the interaction term *X*_1_*X*_2_ (temperature × pH). Both the linear and quadratic terms of temperature were highly significant (*p* < 0.001), underscoring their critical influence on removal efficiency. The linear term for pH was also statistically significant (*p* < 0.01), whereas its quadratic term was not (*p* > 0.4). This suggests that pH affects the system primarily through linear behaviour and synergistic interaction with temperature, rather than exhibiting a pronounced curvilinear effect. The interaction term was statistically significant (*p* < 0.05), confirming a synergistic influence between temperature and pH.

For *q* (mg g^−1^), a similar trend was seen. The significant terms were *X*_1_, *X*_1_^2^, and *X*_1_*X*_2_. Temperature remained the dominant factor, with both linear and quadratic terms showing strong statistical significance (*p* < 0.001). The interaction term was also significant (*p* < 0.05), whereas neither the linear nor the quadratic term of pH was. These findings reinforce the conclusion that adsorption capacity is primarily governed by temperature, with pH exerting an indirect effect through interaction. The inclusion of interaction and quadratic terms is crucial for capturing surface curvature and enhancing model accuracy, particularly for complex adsorption behaviors.

In summary, temperature emerged as the most influential variable in both models, while pH played a secondary but meaningful role through its interaction with temperature. These results validate the use of quadratic models within RSM for process optimisation and provide a robust statistical foundation for predictive modelling in dye adsorption systems. The significant *F*- and *R*^2^-values further support the inclusion of interaction and quadratic terms to capture the system's complexity.

Both response variables, % removal and adsorption capacity (*q*), were strongly affected by temperature and its nonlinear behaviour. The temperature × pH interaction contributed to refining the curvature of the response surface. Although pH alone was not statistically significant in the *q* model, and only marginally significant in the % removal model, its inclusion improved the overall model fit and predictive performance.

To evaluate the adequacy of the fitted quadratic models, residual diagnostics were conducted for both % removal and *q*. The assessment included Q–Q plots, histograms of residuals, residuals *versus* fitted values, and normality testing using the Shapiro–Wilk test.

**Fig. 14:**
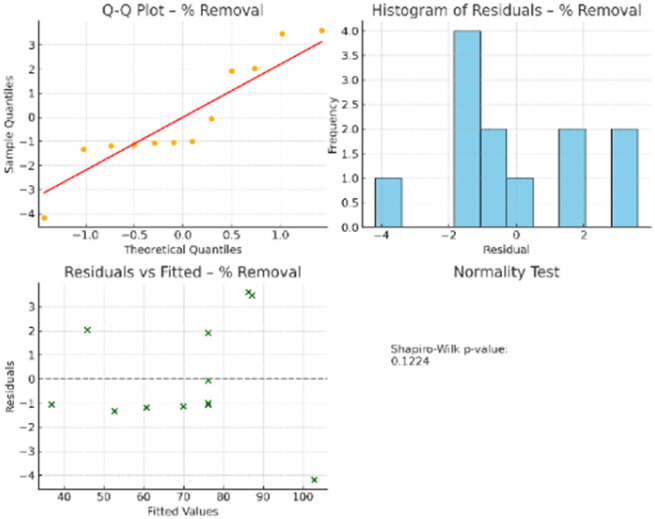
Diagnostic plots for the quadratic model of % removal: Q–Q plot, histogram of residuals, residuals *vs.* fitted values, and Shapiro–Wilk normality test (*p* = 0.1224).

For the % removal model (Fig. 14), the Q–Q plot showed that the residuals closely followed the theoretical normal distribution. The histogram of residuals appeared symmetric, and the residuals *versus* fitted values displayed random scatter without discernible patterns, supporting the assumption of homoscedasticity. The Shapiro–Wilk test returned a *p*-value of 0.1224, exceeding the 0.05 threshold, thus confirming the normality of residuals.

**Fig. 15:**
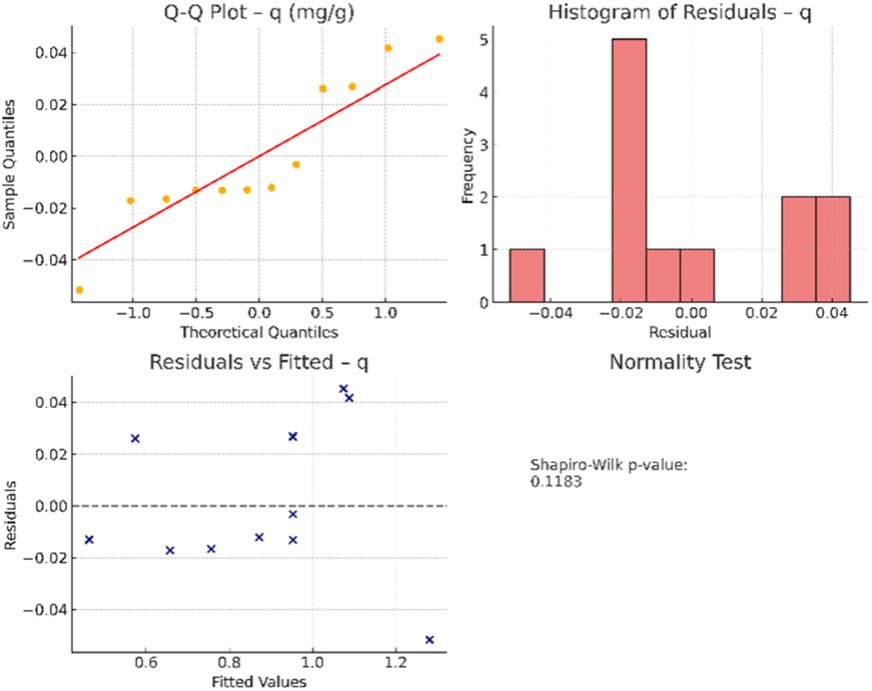
Diagnostic plots for the quadratic model of *q*: Q–Q plot, histogram of residuals, residuals *vs.* fitted values, and Shapiro–Wilk normality test (*p* = 0.1224).

Similarly, the diagnostic plots for the *q* model (Fig. 15) were consistent with the model assumptions. Residuals were normally distributed and randomly dispersed around zero across the range of fitted values. The Shapiro–Wilk test yielded a *p*-value of 0.1183, again supporting the normality assumption.

Collectively, these diagnostic results confirm that both models satisfy the key assumptions of regression analysis: normality, homoscedasticity, and independence of residuals. Meeting these criteria ensures the validity of hypothesis testing, confidence intervals, and optimisation results derived from the models. Accordingly, the response surfaces for both % removal and *q* can be interpreted with high statistical confidence.

Optimisation of the response surface models indicated that both % removal and *q* achieve their maximum predicted values under comparable operational conditions. For % removal, the optimal conditions were 49.6 °C and pH 6.0, yielding a predicted removal efficiency of 99.05%. Similarly, for *q*, the optimum was also found at 49.6 °C and pH 6.0, with a corresponding predicted adsorption capacity of 1.24 mg g^−1^.

**Fig. 16:**
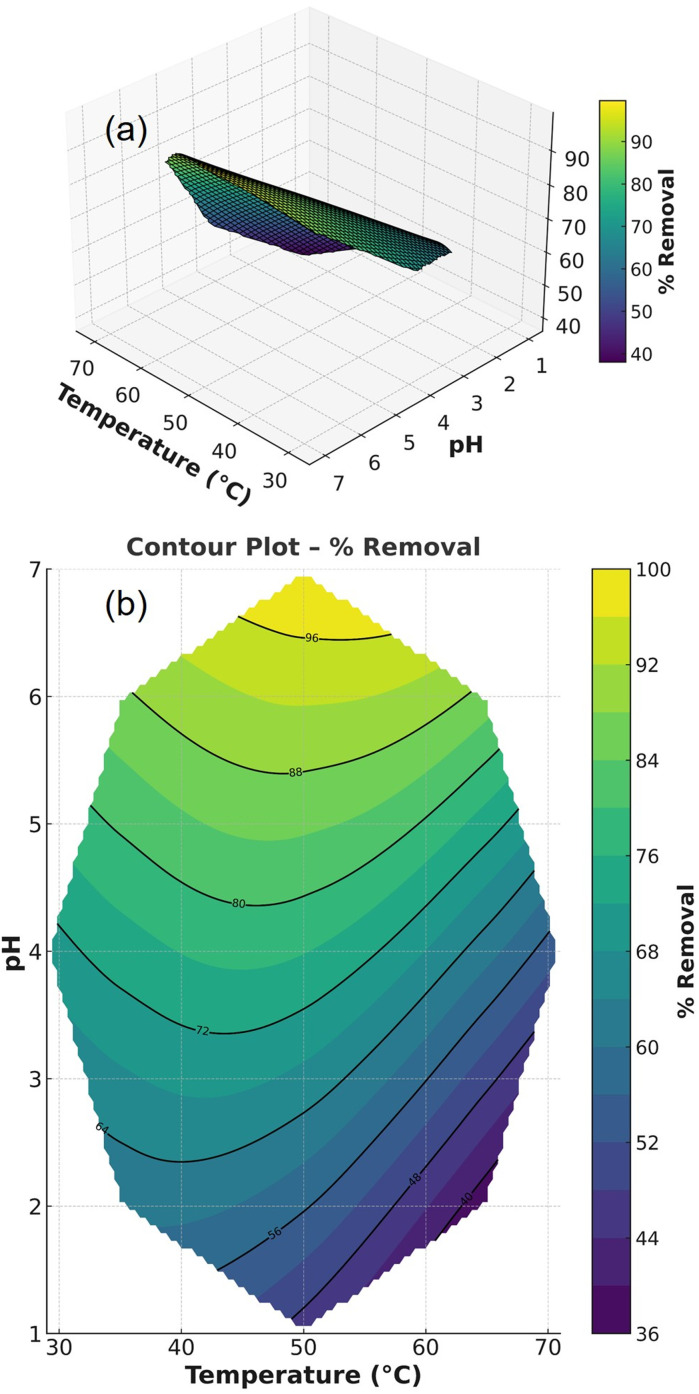
Response surface 3D (a) and contour 2D (b) plots showing the interaction of temperature and pH on % MB removal.

**Fig. 17:**
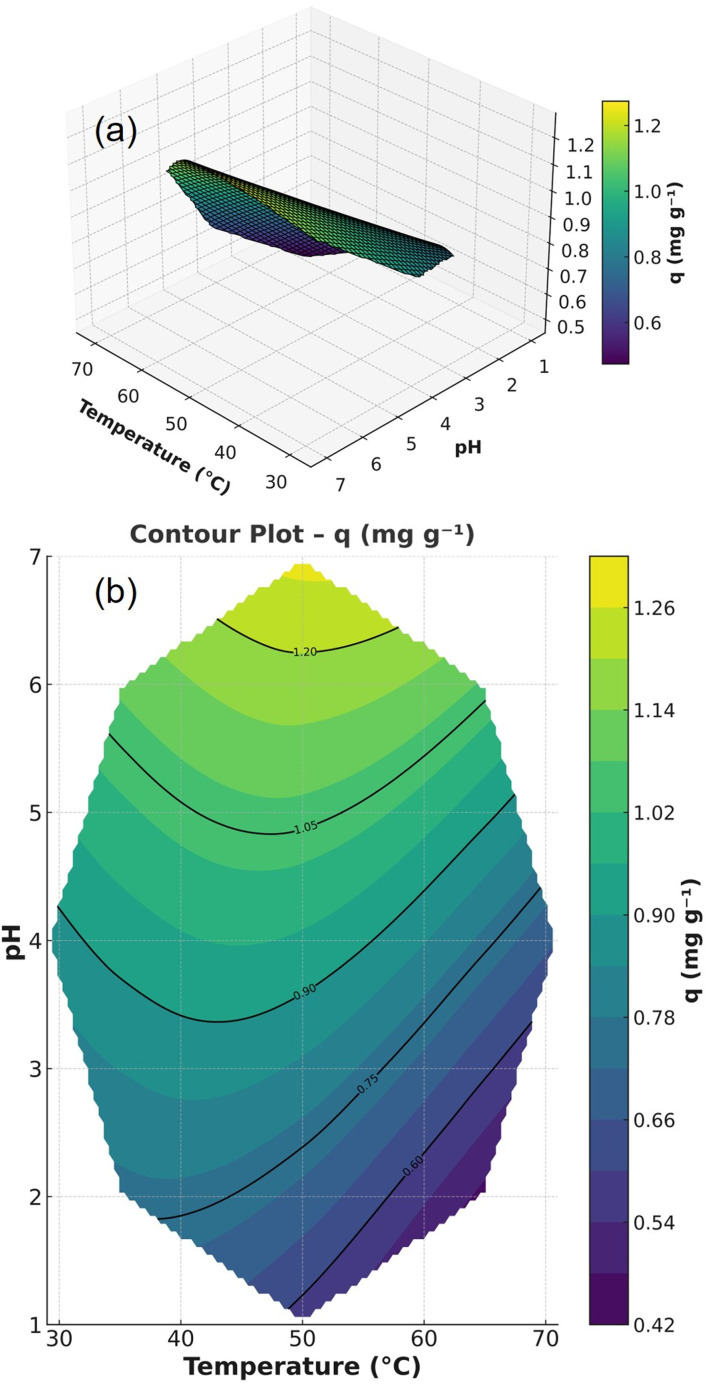
Response surface 3D (a) and contour 2D (b) plots showing the interaction of temperature and pH on *q*.

The three-dimensional response surface plots, along with their corresponding contour diagrams, provide detailed visual insight into the interactive effects of temperature and pH on MB removal efficiency (% removal) and adsorption capacity (*q*, mg g^−1^). These graphical representations illustrate the curvature of the response surfaces and facilitate the identification of optimal operating conditions for both responses. The seen surface curvature and contour gradients emphasise the synergistic influence of temperature and pH, reinforcing their combined impact on system performance (Fig. 16 and 17).

In the response surface for % removal (Fig. 16), a pronounced curvature is seen, particularly along the temperature axis, confirming the strong nonlinear effect of temperature. The response increases with temperature up to an optimal region (∼49.6 °C), beyond which it gradually plateaus, likely due to the saturation of available adsorption sites. The influence of pH is more subtle but plays a role in fine-tuning the response, primarily through its interaction with temperature.

The corresponding contour plot for % removal reinforces these observations by delineating zones of high efficiency in the central region of the design space. A region of maximum removal efficiency (>95%) is centered around moderate to high temperatures (approximately 48–55 °C) and slightly acidic to neutral pH values (5.5–6.5). The presence of elongated, diagonally oriented elliptical contour lines highlights a significant interaction between temperature and pH, where small changes in pH at a given temperature can markedly affect the removal efficiency.

Similarly, the response surface plot for q exhibits a curved topography (Fig. 17), with a distinct maximum occurring at intermediate levels of both temperature and pH. Temperature again emerges as the dominant factor, while pH contributes to enhancing the response primarily through its synergistic interaction with temperature. The contour lines for *q* are more symmetrical and uniformly spaced, indicating a smoother gradient and suggesting broader operability within the optimal region.

The contour plot for *q* identifies a high-capacity adsorption zone (*q* > 1.1 mg g^−1^) within a similar operational window, moderate temperatures (around 50 °C), and near-neutral pH values (6.0–6.5). In contrast to the % removal plot, the contour lines here are more concentric and evenly distributed, suggesting that *q* responds more uniformly to variations in either factor, with less pronounced interaction effects.

The overlap between high-response regions in both contour plots highlights a shared optimal range, demonstrating the feasibility of a unified set of operating conditions that simultaneously maximises % removal and *q*. These visual representations not only validate the predictive strength of the quadratic regression models but also facilitate the direct identification of robust process windows, making them valuable tools for practical process control and scale-up.

Collectively, these graphical tools reinforce the statistical conclusions derived from the quadratic models and allow for intuitive visualization of factor interactions and response behaviour. The response surfaces confirm the adequacy of model fit and reveal the sensitivity of the process to changes in operating parameters, supporting robust and flexible process design.

Ultimately, the integration of these findings enables effective model-based optimisation and provides mechanistic insight into the adsorption behaviour under variable conditions. The developed models offer a reliable framework for predicting adsorption performance and serve as a foundation for advanced system design and scale-up in dye removal applications.

Although the ANOVA results for the quadratic model indicate that pH was not a statistically significant main factor (*p* > 0.05), this does not imply that pH lacks influence within the adsorption system. Experimental evidence shows that at pH 6, the ANCSH5 adsorbent achieved higher % removal, which can be attributed to the physicochemical properties of its surface functional groups.

At this pH, the surface of ANCSH5 exposes a greater density of active binding sites, including deprotonated carboxyl groups and aromatic regions capable of π–π stacking with MB^+^ molecules. These structural features enhance the adsorbent's affinity and capacity for dye uptake, particularly under slightly acidic to neutral conditions.

This mechanistic understanding aligns with the seen experimental enhancement in % removal at pH 6, despite the statistical model assigning low standalone significance to pH. The key lies in the interaction term: ANOVA revealed that the temperature × pH interaction was statistically significant (*p* < 0.05), indicating that the influence of pH becomes more pronounced when combined with thermal effects.

In contrast, other materials such as ANCSA10 and CPH did not exhibit similar performance at pH 6, likely due to differences in surface functionality and active site availability. This highlights the material-dependent nature of pH sensitivity in adsorption processes.

Thus, the apparent contradiction between statistical significance and experimental trends can be resolved. While pH may not exert a uniform linear influence across the design space, it plays a critical interactive role under specific conditions. The temperature remains the dominant variable, but pH significantly enhances adsorption performance when acting in synergy, particularly in materials like ANCSH5 with favorable surface chemistry.

## 4 Conclusions

This study demonstrated the green synthesis of ANC from CPH *via* acid and alkaline activation for MB removal. Among the prepared materials, ANCSH5 (NaOH, 5 M) exhibited the best performance due to extensive delignification, enhanced graphitization, and abundant oxygenated surface functionalities, as confirmed by FTIR, SEM-EDX, BET, XRD, and Raman analyses. The improved surface polarity further promoted MB adsorption at pH 6 through electrostatic interactions, hydrogen bonding, and π–π stacking. Adsorption followed pseudo-second-order kinetics, while equilibrium data were best fitted by the Freundlich, Sips, Toth, and Redlich–Peterson models (*R*^2^ > 0.999). Process optimisation using CCD-RSM identified optimal conditions (49.6 °C, pH 6.0), achieving 99.05% MB removal.

Importantly, ANCSH5 reached a competitive capacity (100 mg g^−1^, isotherm-derived) under realistic operating conditions (*C*_0_ = 5–30 mg L^−1^, pH 6, 35 °C, dosage = 4 g L^−1^), unlike other biomass-derived carbons that report higher *q*_max_ values only under extreme concentrations or excessive dosages. This distinctive performance arises from the hemicellulose-rich composition of CPH, which under alkaline activation promotes delignification, partial graphitization, and tailored surface chemistry. Together with validated statistical optimisation, these features establish CPH-derived ANC as a sustainable, low-cost, and industrially competitive adsorbent for dye remediation aligned with circular economy strategies. Overall, the synthesis route not only provides a high-performing adsorbent but also represents a greener alternative by coupling agro-waste valorisation with reduced energy and chemical demands, in contrast to conventional biomass- or fossil-based ACs.

This study did not include high-resolution electron microscopy (HRTEM) with particle-size analysis, density functional theory (DFT) calculations, or standardised ecotoxicity bioassays due to limited access to instrumentation. Nevertheless, the physicochemical characterisation and adsorption performance suggest that the materials may possess nanometric features, as supported by BET, SEM-EDX, XRD, Raman, and FTIR analyses, together with advanced isotherm and kinetic modelling. Additional high-resolution studies will be required to conclusively confirm their nanostructured nature. Future work will integrate HRTEM with quantitative particle sizing, DFT simulations of MB-surface interactions, and OECD-compliant ecotoxicity assays (*e.g.*, *Daphnia magna* immobilisation and algal growth inhibition) to provide a more comprehensive understanding of structure–property relationships and environmental safety.

## Author contributions

Zenaida Guerra-Que: conceptualization, supervision, project administration, writing – review & editing, funding acquisition. Katia S. López-Margalli: methodology, investigation, data curation, writing – original draft. Juan Manuel Urrieta-Saltijeral: formal analysis, validation, software, visualization. Adib Abiu Silahua-Pavón: experimental design, response surface methodology analysis, resources. Héctor Martínez-García: characterisation (FTIR, SEM-EDX), data interpretation, writing – review & editing. Pedro García-Alamilla: Lignocellulosic analysis, Material preparation, Supervision. Gerardo E. Córdova-Pérez: XRD and raman analysis, instrumentation, technical support. Juan Carlos Arévalo-Pérez: SEM-EDX supervision, laboratory facilities, method validation. José Gilberto Torres-Torres: visualization, graphical abstract design, review, and critical revisions.

## Conflicts of interest

The authors declare no conflicts of interest.

## Data Availability

The data supporting the findings of this study are available within the article. Additional datasets generated and analyzed during the current study are available from the corresponding author upon request.
